# Multi‐Omic Profiling of T Cell‐Mediated Rejection After Kidney Transplantation Reveals B Cell Receptor Repertoire Expansion and Its Prognostic Relevance

**DOI:** 10.1096/fj.202502448RR

**Published:** 2025-12-30

**Authors:** He Zhang, Di Zhang, Yuhong Hu, Hao Zhang, Zijian Zhang, Xiaopeng Hu

**Affiliations:** ^1^ Department of Urology, Beijing Chaoyang Hospital, Capital Medical University Beijing China; ^2^ Institute of Urology, Capital Medical University Beijing China; ^3^ Chinese Academy of Medical Sciences & Peking Union Medical College Beijing China

**Keywords:** B cell receptor, immune repertoire, kidney transplantation, plasma cell, T cell‐mediated rejection

## Abstract

This study explores the immune repertoire landscape of B cell receptor (BCR) in renal allografts with T cell‐mediated rejection (TCMR) to identify new therapeutic targets due to the limitations of current immunosuppressive treatments. BCR repertoires were constructed from both bulk and single‐cell RNA‐seq data utilizing the TRUST4 algorithm. The study revealed a significant expansion of the BCR repertoire, particularly Immunoglobulin G in TCMR compared to stable renal function across three independent renal transplant cohorts. By integrating multi‐omics data from various datasets including bulk transcriptomic data, single‐cell transcriptome profiles, cell line data, and immunofluorescence of renal allograft biopsies, the infiltrated plasma cell was identified as the primary holder of BCRs in renal allografts with TCMR, serving as an independent risk factor for prognosis. Additionally, *MEI1* was discovered as a novel BCR‐related key gene in TCMR, upregulated during plasma cell maturation and contributing to antibody secretion. This study first delineated the BCR repertoire landscape in TCMR, highlighting the crucial role of BCR, allograft‐infiltrated plasma cells, and *MEI1* in TCMR, thus offering novel therapeutic targets for improving allograft outcomes.

## Graphical abstract

This study integrates bulk and single‐cell RNA‐seq data to reveal significant B cell receptor (BCR) repertoire expansion, especially IgG isotype amplification, in T cell‐mediated rejection (TCMR) after kidney transplantation. Multi‐omic integration identifies plasma cells as the main BCR carriers in graft tissue and *MEI1* as a key regulator promoting antibody secretion. These findings highlight plasma cell infiltration and *MEI1*‐driven antibody production as crucial determinants of TCMR prognosis and potential therapeutic targets.

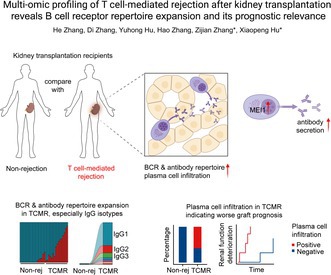

## 1 Introduction

T cell‐mediated rejection (TCMR) is a severe complication after transplantation, greatly compromising allograft prognosis [1]. Despite advancements in immunosuppression, TCMR was diagnosed in 5% ~ 15% of recipients in the first year after kidney transplantation [2]. Moreover, subclinical TCMR is detected in 11%–30% of early protocol biopsies within 12 months post‐transplantation and is also associated with donor‐specific antibodies, chronic allograft nephropathy, and graft loss [3]. Current options for treating TCMR in clinical practice are increasing doses of immunosuppression, high‐dose steroids, and thyroglobulin. However, these non‐targeted strategies often show inadequate treatment response, resulting in either persisting TCMR or recurrence in many patients [4]. A recent meta‐analysis involving 1255 participants from 12 studies demonstrated that TCMR persists in over one‐third of cases even after appropriate treatment for TCMR over a period of 2–9 months [5]. Large biopsy cohort studies demonstrated that persistent or recurrent TCMR led to increased 2–3 fold risks of chronic graft injury and graft loss [6]. Therefore, there is still an urgent need to investigate the complex mechanisms underlying TCMR and explore new targeted interventions.

The immune repertoires, comprising the B cell receptor (BCR) and T cell receptor (TCR), are essential molecules located on the surfaces of B and T cells, facilitating antigen‐specific recognition and modulation of immune responses [7]. Through special mechanisms such as V(D)J recombination and somatic hypermutation, BCR and TCR generate an estimated 10^15^–10^20^ distinct clonal types. These BCRs and TCRs are collectively referred to as the immune repertoire [8]. Research on immune repertoires has gained increasing attention in many fields, including infectious [9] diseases, allergy [10], autoimmunity [11], and cancer immunotherapy [12, 13]. In the field of organ transplantation, investigating the immune repertoire will enhance our understanding of the mechanisms of rejection, facilitating the identification of individualized biomarkers and the development of therapeutic targets. Our previous study elucidated the enhanced TCR repertoire in renal allografts with TCMR and identified novel candidates for the diagnosis and targeted treatment. While BCR inside the allograft has always been a neglected factor in TCMR with very few reports [14]. Carpio et al. found that CD20‐positive B‐cell infiltrations were associated with T‐cell mediated rejection in for‐cause biopsy samples [15]. Schiffer et al. investigated 67 randomly selected TCMR biopsy cases; 56 showed significant B‐cell infiltration, yet the mechanisms underlying this phenomenon remain unclear [16].

Given the urgent need for searching undiscovered mechanisms and new therapeutic targets for TCMR, this study conducted the first exploration of the BCR landscape in renal allografts with TCMR across large cohorts. Taking advantage of the TRUST4 algorithm, BCR repertoires were constructed from the raw base sequences of both bulk and single‐cell RNA‐seq data. Our results showed a prominent expansion of the BCR repertoire, especially Immunoglobulin G (IgG), in TCMR compared to stable renal function (STA) in two independent renal transplant cohorts. Integrating multi‐omics data from multiple datasets (including bulk transcriptomic online data from human cohorts, single‐cell transcriptome open resource profiles, cell line data, and immunofluorescence of renal allograft biopsies), the infiltrated plasma cell is identified to be the dominating holder of BCRs in renal allografts with TCMR and serves as an independent risk factor for the prognosis of allografts. In addition, *MEI1* was identified as a novel BCR‐related key gene in TCMR. *MEI1* is up‐regulated during plasma cell maturation and contributes to antibody secretion, offering a new potential therapeutic target for plasma cell‐related diseases. Overall, our study for the first time exhibited the BCR repertoire landscape in TCMR and emphasized the important role of BCR, allograft‐infiltrated plasma cells, and *MEI1* in TCMR from large patient cohorts, offering novel therapeutic targets.

## 2 Methods

### 2.1 Data Source and Processing

Table 1 summarizes detailed information on the eleven datasets employed in this study. In brief, one RNA‐seq dataset (GSE131179 [17]) comprises 18 TCMR biopsy samples and 16 STA biopsy samples, another RNA‐seq dataset (GSE232825 [18]) contains 17 acute TCMR, 7 acute antibody‐mediated rejection (aABMR), 17 chronic acute ABMR (caABMR), and 18 no‐rejection renal allograft biopsies (excluding mixed rejection, BK virus nephropathy, and other diagnoses). Five microarray datasets include two TCMR and ABMR datasets (GSE36059 [19, 20] and GSE129166 [21]), two TCMR datasets (GSE72925 [22, 23] and GSE25902 [24]), and one kidney transplant dataset (GSE21374 [25]) with long‐term follow‐up. The single‐cell RNA‐seq dataset PRJNA974568 [26] includes 15 acute cellular rejection(ACR) biopsy samples and 6 non‐rejection (NR) biopsy samples. Plasma cell datasets (GSE175384 [27] and GSE175385 [27]) are based on bulk and single‐cell RNA‐seq separately. In our clinical study, the inclusion criteria for screening samples of TCMR tissues were as follows: [1] Patients aged between 18 and 65 years; [2] Patients hospitalized between January 1, 2017, and December 31, 2021, with for‐cause biopsies performed during hospitalization; [3] Patients diagnosed with TCMR based on pathological biopsy. STA patients were selected by matching clinical characteristics with TCMR patients. The STA tissue samples included in this study met the following criteria: [1] Patients aged between 18 and 65 years; [2] Patients hospitalized between January 1, 2017, and December 31, 2021, at our institution, with biopsies performed during hospitalization; [3] Pathological findings indicating no rejection. A total of twenty kidney biopsy samples (ten TCMR and ten STA) were obtained, with final diagnoses determined by two pathologists. Informed consent was obtained from all patients, and ethical approval was granted by the hospital's ethics committees. The clinical characteristics of the above patients are shown in Table S1. The *p* values of clinical characteristics between the TCMR and STA groups were all above 0.05. Relapse of renal function deterioration was defined as an elevation of blood creatinine level above 50% of the baseline.

**TABLE 1:** Information of twelve datasets included in this study.

Datasets	Platforms	Species	Tissues	Sample size	Applications	References (PMID)
GSE131179	Illumina HiSeq 2500	*Homo sapiens*	Kidney biopsy	16 TCMR 18 STA	Construction immune repertoire applying TRUST 4 algorithm and bulk dataset analysis	32 102 984
GSE232825	Illumina HiSeq 2500	*Homo sapiens*	Kidney biopsy	17 TCMR 7 aABMR 17 caABMR 18 STA	Construction immune repertoire applying TRUST 4 algorithm and bulk dataset analysis	38 040 290
GSE72925	Affymetrix Human Genome U133 Plus 2.0 Array	*Homo sapiens*	Kidney biopsy	26 TCMR 73 STA	Bulk datastes analysis and graft dignosis analysis	31 632 976
GSE25902	Affymetrix Human Genome U133 Plus 2.0 Array	*Homo sapiens*	Kidney biopsy	24 TCMR 96 STA	Bulk dataset analysis	21 881 554
GSE36059	Affymetrix Human Genome U133 Plus 2.0 Array	*Homo sapiens*	Kidney biopsy	35 TCMR 281 STA 65 ABMR 22 Mix Rejection	Bulk dataset analysis	25 377 077; 24 700 874; 23 356 949
GSE129166	Affymetrix Human Genome U133 Plus 2.0 Array	*Homo sapiens*	Kidney biopsy and Peripheral blood	13 TCMR 60 STA 15 ABMR 7 Mix Rejection	Bulk dataset analysis	31 378 695
GSE175384	Illumina NextSeq 500	*Homo sapiens*	Kidney biopsy	16 Healthy Adults Plasma cell samples	Bulk dataset analysis	34 133 718
GSE175385	Illumina NextSeq 500	*Homo sapiens*	Kidney biopsy	3 Healthy Adults Plasma cell samples	Single cell RNA‐seq analysis	34 133 718
PRJNA974568	Illumina NovaSeq 6000	*Homo sapiens*	Kidney biopsy	15 TCMR 6 STA	Construction immune repertoire applying TRUST 4 algorithm and Single cell RNA‐seq analysis	37 227 784
Chaoyang Hospital cohort	Immunofluorescent staining	*Homo sapiens*	Kidney	10 TCMR 10 STA	Protein and graft survival analysis	This study
GSE21374	Affymetrix Human Genome U133 Plus 2.0 Array	*Homo sapiens*	Kidney biopsy	282 KT with follow‐up	Graft survival analysis	20 501 945

Abbreviations: aABMR: Acute Antibody‐Mediated Rejection; caABMR: Chronic Active Antibody‐Mediated Rejection; KT: Kidney Transplantation; STA: stable renal function; TCMR: T Cell Mediated Rejection.

### 2.2 B‐Cell Receptor Repertoire Construction and Analysis

TRUST4 algorithm [28] (version 1.0.12) was applied to reconstruct BCR repertoires from the FASTQ file of bulk RNA‐seq and single‐cell RNA‐seq data, which is effective in reconstructing immune receptor repertoires. Following standard algorithm pipelines utilizing RNA‐seq data of GSE131179 and PRJNA974568, output data including BCR repertoire information was obtained. Then, we analyzed the samples statistically utilizing the number of clonotypes (absolute abundance) and the number of clonotypes per thousand CDR3 reads (CPK) that represented the diversity of the sample. Further analysis will be conducted based on the division of different chains in heavy chain and light chain. Since the antibody constant region is important for downstream immune signaling, the heavy chain is subdivided into Immunoglobulin Heavy Constant Alpha (IGHA), Immunoglobulin Heavy Constant Delta (IGHD), Immunoglobulin Heavy Constant Epsilon (IGHE), Immunoglobulin Heavy Constant Gamma (IGHG), and Immunoglobulin Heavy Constant Mu (IGHM). The IGHG is subdivided into IGHG1, IGHG2, IGHG3, and IGHG4, while the IGHA is subdivided into IGHA1 and IGHA2. In the statistical analysis of Immunoglobulin heavy chain variable region (IGHV) genes, genes with low expression in at least 10% of the TCMR samples were excluded. Subsequently, we obtained candidate genes for further analysis from the following criteria: [1] Strong correlations (*R* > 0.7) with the number of IGHG clonotypes [2] Strong correlations (*R* > 0.7) with CPK of IGHG [3] Differential expression genes (Adjusted *p* < 0.05, Hochberg correction method).

### 2.3 Bulk Transcriptome Data Analysis

All RNA‐Seq and microarray data were standardized, which was provided by the authors in the GEO database (https://www.ncbi.nlm.nih.gov/geo). Immune cell infiltration was evaluated by the CIBERSORT‐ABS algorithm [29]. Then, gene set enrichment analysis (GSEA) was performed (based on the Human MSigDB database v2022.1.Hs) [30].

### 2.4 Single‐Cell RNA‐Seq Data Analysis

The single‐cell data were analyzed utilizing the Seurat R package (version 4.2.1; https://satijalab.org/seurat/). Quality control, initial clustering, and annotations were conducted following the methods described in the original research. Visualization of cells and clusters was achieved using the uniform manifold approximation and projection method. Subsequently, B cells and plasma cells were selected for further re‐clustering and re‐annotation based on the expression levels of *MEI1* across all cell types. The determination of the number of top components was guided by the elbow plot. The resolution parameters were set to generate enough clusters to capture major biological variability while avoiding redundant clustering. The GSEA was performed by comparing gene expression profiles of cells with *MEI1* positive expression and negative expression. Gene sets from the Human MSigDB database (v2022.1.Hs) were used as the reference for GSEA in single‐cell analysis. The pseudo‐time trajectory analysis of plasma cells was performed using Monocle 2 [31].

### 2.5 Immunofluorescent Staining

Paraffin‐embedded slides of kidney tissues obtained from our clinical cohort were deparaffinized and antigenic repair was performed using citric acid. Primary antibodies include IRF4 (Proteintech), CD3 antibody (ZSGB‐BIO, China), and CD8 antibody (ZSGB‐BIO, China). Triple immunofluorescence (IF) staining was carried out using the Tyramide Signal Amplification method. Nuclear staining was performed with DAPI (C0060, Solarbio, China). The imageries of the immunofluorescence were captured using an Olympus BX53 microscope (Olympus, Tokyo, Japan). Images were analyzed using the ImageJ software (https://imagej.net/ij/) on the Fiji platform.

### 2.6 Graft Survival Analyses

To evaluate the effects of plasma cell infiltration and expression of *MEI1* on long‐term allograft survival, the GSE21374 dataset containing graft survival data of kidney transplant patients was utilized. Kaplan–Meier curves were generated using the survival R package (version 3.5–3; https://github.com/therneau/survival), and a multivariate Cox regression analysis was conducted to examine the relationship between gene expression and recipient rejection status.

### 2.7 CRISPR‐Cas9 Mediated Genome Knockout

CRISPR‐Cas9 mediated genome editing was performed using a structured and systematic approach. In brief, single‐guide RNA sequences targeting the *MEI1* coding sequence were designed utilizing the online tool provided by the Broad Institute (https://portals.broadinstitute.org/gppx/crispick/public). A total of four single‐guide RNAs were designed (Table S2) and the one with the best knockout efficiency was utilized for subsequent experiments. Following the design, oligonucleotide pairs were annealed and ligated into an Esp3I‐linearized, modified Lenti‐U6‐gRNA‐Cas9‐Puro‐GFP plasmid, a derivative of the PLKO.1‐puro vector. The recombinant plasmid along with the lentiviral packaging plasmids, PsPAX2 and PmD2.G, were co‐transfected into HEK293T cells using the Lipofectamine 3000 transfection reagent (Life Technologies), following the manufacturer's protocol. Viral particles were harvested from the supernatant. Subsequently, logarithmic phase LP‐1 cells were transduced with the viral suspension at a 1:1 ratio. The medium was replaced after 24 h to remove unincorporated virus particles, and the cells were cultured under expansion conditions. Selection of stably transduced cells was initiated 3 days post‐transduction by adding puromycin at a concentration of 5 μg/mL. The medium was refreshed after 36 h, and selection was repeated 24 h later. Knockout efficiency was assessed via quantitative polymerase chain reaction.

### 2.8 Cell Culture and Treatment

The human plasma cell leukemic cell line LP‐1 (EK‐Bioscience) was cultured in Iscove‐modified Dulbecco's medium containing 10% fetal calf serum, 1% penicillin, and streptomycin and maintained at 37°C in 5% CO_2_. The cells were tested to be free of mycoplasma contamination. The LP‐1 cells were stimulated by lipopolysaccharide (LPS, Sigma‐Aldrich) with different concentrations (0, 10, 100, 1, 10 μg/mL) for 48 h.

### 2.9 Quantitative Polymerase Chain Reaction Analysis

Total RNA of cells was extracted and reverse transcribed to cDNA using RevertAid First Strand cDNA Synthesis Kit (Thermo Fisher Scientific). Relative expression of the target gene was analyzed using the 2^−ΔΔCT^ method with GAPDH as the reference gene. The primers are listed as follows:

*MEI1*, forward 5′‐ACCCTCTTGGATGCTGGAGA‐3′, reverse 5′‐TGCAGAACATCACCCACCTC‐3′;

GAPDH, forward 5′‐ACCAGGGCTGCTTTTAACTCTG‐3′, reverse 5′‐TGGGTGGAATCATATTGGAACAT‐3′.

### 2.10 Measurement of Cell Proliferation and Viability

Cell proliferation was measured using the cell counting kit‐8 (CCK8) assay. Briefly, 100 μL of cell suspension (5000 cells per well) was added per well in a 96‐well plate and then cultured for 24 h. After treatment, 10 μL of CCK‐8 solution (ApexBio) was added to each well and incubated for 2 h. Then, the absorbance at 450 nm was measured using a microplate reader (Multiskan FC, Thermo Fisher Scientific).

Cell viability was further evaluated using a trypan blue exclusion assay. Stably transduced MEI1 knockout and control cells were harvested, stained with 0.4% trypan blue solution (ApexBio), and counted using the hemocytometer (Multiskan FC, Thermo Fisher Scientific). The percentage of viable cells was calculated as the number of trypan blue–negative cells divided by the total number of cells.

### 2.11 Determination of Antibody Levels

The antibody levels in the culture medium were detected using the enzyme‐linked immunosorbent assay kit (Novus Biologicals, Bio‐Techne).

### 2.12 Statistical Analyses

Statistical analyses were conducted using R software (version 4.2.1). The normality of the data was assessed, and unpaired Student t‐tests, one‐way ANOVA, and Pearson correlation were utilized for the analysis of normally distributed data. For non‐normally distributed data, Kruskal−Wallis, Wilcoxon test, and Spearman correlation were employed. Results with a *p*‐value below 0.05 were considered statistically significant.

## 3 Results

### 3.1 Landscape of BCR Repertoire in Renal Allografts With TCMR and STA

**FIGURE 1:**
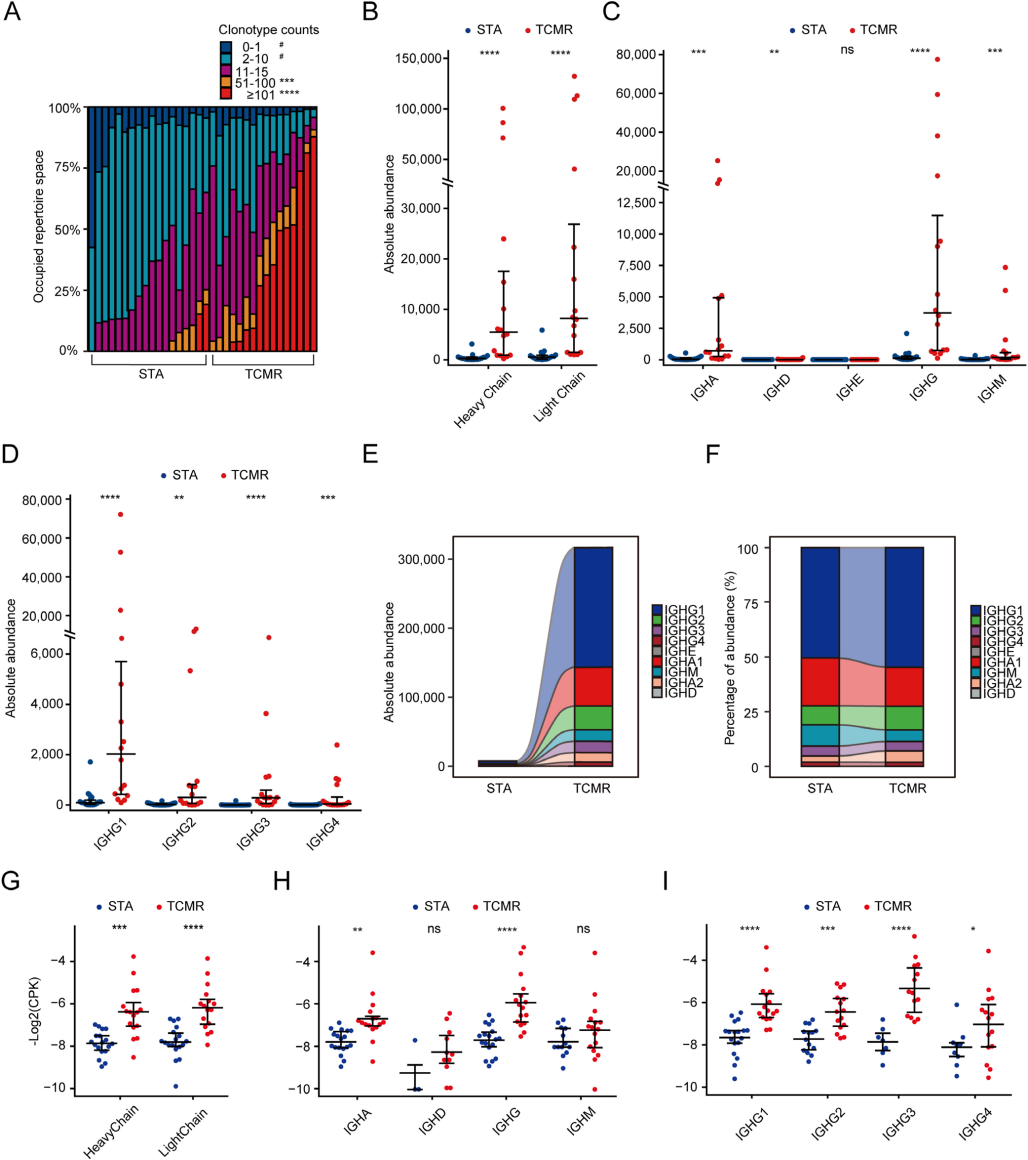
Construction and analysis of B cell receptor repertoire in bulk dataset GSE131179. (A) Histogram plot shows the clonality of each sample with specific counts. Beeswarm plots demonstrate the comparison of the absolute abundance between the T cell‐mediated rejection (TCMR) group and the stable renal function (STA) group in heavy chain and light chain (B), heavy chain subdivision including IGHA, IGHD, IGHE, IGHG, IGHM (C) and IGHG subdivision including IGHG1, IGHG2, IGHG3, IGHG4 (D). Alluvial diagrams represent the differences between the TCMR group and the STA group for absolute abundance (E) and percentage of absolute abundance (F). Beeswarm plots demonstrate the comparison of the clonotypes per thousand CDR3 reads (CPK) between the TCMR group and the STA group in heavy chain and light chain (G), heavy chain subdivision including IGHA, IGHD, IGHE, IGHG, IGHM (H) and IGHG subdivision including IGHG1, IGHG2, IGHG3, IGHG4 (I). (*) indicates that the TCMR group is higher than the STA group, and (#) indicates that the STA group is significantly greater than the TCMR group. *p < 0.05; **p < 0.01; ***p < 0.001; ****p < 0.0001. Data are presented as mean ± SEM.

The BCR repertoire was constructed using the standard pipeline of the TRUST4 algorithm from bulk RNA‐seq data (GSE131179). First, BCR clonalities were categorized into 5 groups according to their clonotype counts, and the proportion of these groups in each sample is displayed (Figure 1A). The BCR repertoire of TCMR patients was primarily dominated by clonally expanded BCRs (clonotype count 51–100, *p* < 0.001 and ≥ 101 copies, *p* < 0.0001), while STA patients had a significantly higher percentage of low counts (clonotype count 0–1, *p* < 0.05 and ≥ 101 copies, *p* < 0.0001; Figure 1A) and Figure S1A. The total BCR abundance in TCMR is expanded about 38‐fold compared to STA. The absolute abundance of heavy chain and light chain in the TCMR group was found to be significantly greater than that of the STA group (*p* < 0.0001, Figure 1B). Subsequently, a more detailed analysis was conducted by further subdividing the heavy chain into IGHA, IGHD, IGHE, IGHG, and IGHM. It was observed that the absolute abundance of IGHA (*p* < 0.001), IGHD (*p* < 0.01), IGHG (*p* < 0.0001), and IGHM (*p* < 0.001) in the TCMR group was significantly higher compared to the STA group (Figure 1C). In comparison to the other four subgroups, a significant increase in the absolute abundance of IGHG was observed in the TCMR group. Consequently, our focus shifted towards examining changes within the IGHG subclass. The absolute abundance of IGHG1 (*p* < 0.0001), IGHG2 (*p* < 0.01), IGHG3 (*p* < 0.0001), and IGHG4 (*p* < 0.001) in the TCMR group surpassed that of the STA group (Figure 1D). The alluvial diagram depicting absolute abundance (Figure 1E) further illustrates a notable increase in IGHG1 within the TCMR group compared to the STA group with each BCR subtype represented by a distinct color. Consistently, the percentage of abundance (Figure 1F) also indicates a rise in the proportion of IGHG1 at the same time. Subsequently, the clonotypes per thousand CDR3 reads (CPK, representing the BCR diversity) was applied to evaluate the diversity of the BCR in GSE131179. Similarly, we observed an increase in diversity in both the heavy chain (*p* < 0.001) and light chain (*p* < 0.0001, Figure 1G), However, upon further segmentation of the heavy chain, we only observed a notable increase in the diversity of IGHA (*p* < 0.01) and IGHG (*p* < 0.0001, Figure 1H). In Figure 1H, IGHE did not display the diversity due to almost no detection in all samples. In the subgroups of IGHG, the diversity of IGHG1 (*p* < 0.0001), IGHG2 (*p* < 0.001), IGHG3 (*p* < 0.0001), and IGHG4 (*p* < 0.05) also exhibited a significant increase in TCMR group compared to STA group (Figure 1I).

The BCR/IgG signature is classically associated with ABMR. To further investigate the similarities and differences in BCR repertoire in renal allografts with TCMR and ABMR, we employed another renal transplantation cohort (GSE232825) with 17 acute TCMR, 7 acute ABMR (aABMR), 17 chronic acute ABMR (caABMR), and 18 no‐rejection renal allograft biopsies (excluding mixed rejection, BK virus nephropathy, and other diagnoses). Both TCMR and caABMR, but no aABMR, exhibited a statistically significant increase in both total BCR abundance, heavy and light chain, and IGHG expression compared to the no rejection biopsy samples Figure S2A–C. In terms of specifically expanded IGHV genes, there is an obvious difference between TCMR and caABMR Figure S2D.

### 3.2 BCR Is Predominantly Expressed by Plasma Cells in Renal Allografts

**FIGURE 2:**
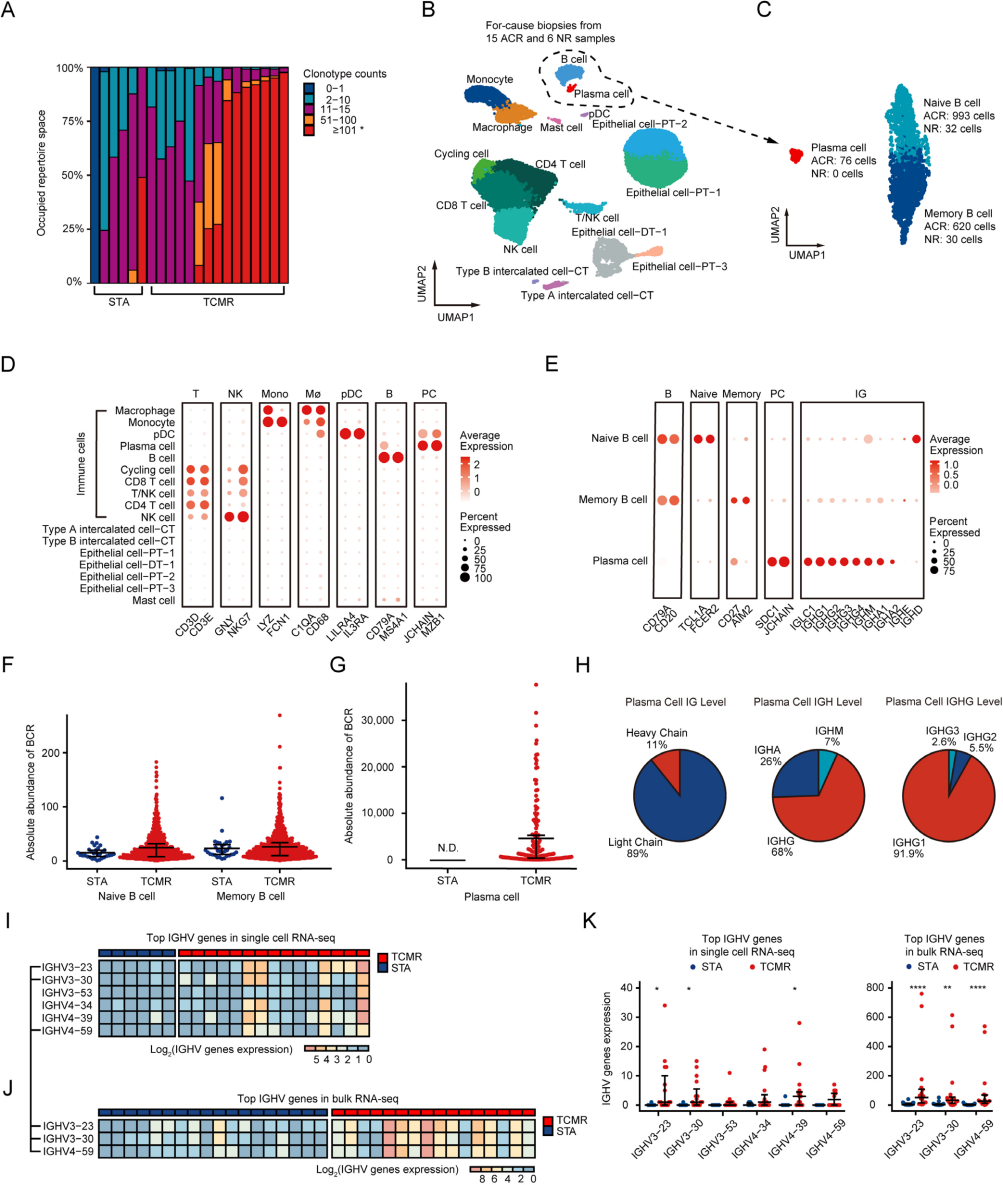
Construction and analysis of B cell receptor repertoire in single‐cell dataset PRJNA974568. (A) Histogram plot shows the clonality of each sample with specific counts. (B) The uniform manifold approximation and projection (UMAP) plot from 15 acute cellular rejection biopsy samples and 6 non‐rejection biopsy samples in different cell types. (C) UAMP plot of subtypes of Naive B, Memory B cells, and Plasma cells. Dim plots demonstrate gene expression in different cell types (D) and subtypes of Naive B, Memory B cells, and Plasma cells (E). Beeswarm plots demonstrate the comparison of the absolute abundance between the T cell‐mediated rejection (TCMR) group and the stable renal function (STA) group in Naive B and Memory B cells (F), and Plasma cells (G). (H) Circular graphs describe the proportion of heavy chain and light chain, heavy chain subdivision including IGHA, IGHD, IGHE, IGHG, IGHM, and IGHG subdivision including IGHG1, IGHG2, IGHG3, IGHG4. Heatmaps display each sample log2 expression of Top IGHV genes in single‐cell RNA‐seq (I) and bulk RNA‐seq (J) datasets. (K) Beeswarm plots show the expression of Top IGHV genes between the TCMR group and the STA group in single‐cell RNA‐seq and bulk RNA‐seq datasets. N.D. indicates not detectable. * *p* < 0.05; **p < 0.01; ****p < 0.0001. Data are presented as mean ± SEM.

The single‐cell RNA‐seq data obtained from project PRJNA974568 was utilized to examine the B‐cell receptor repertoire. The analysis revealed that BCRs still significantly expanded in the biopsies with TCMR (clonotype count ≥ 101 copies, *p* < 0.05; Figure 2A) and Figure S1B. Next, the data was employed to investigate BCR repertoire under more detailed cell subpopulations. Following quality control measures, 17 distinct cell types were identified through clustering and annotation of 15 acute cellular rejection (ACR) and 6 non‐rejection (NR) biopsy samples (Figure 2B) and Figure S3. Subsequently, B cells and plasma cells were selected for further analysis. Based on the ACR and NR classifications, a total of 993 ACR and 32 NR cells were identified in naive B cells, 620 ACR, and 30 NR cells in memory B cells, while plasma cells only detected 76 ACR cells (Figure 2C). In the analysis of marker gene‐based groupings (Figure 2D), particular attention was given to the expression patterns of antibody‐related genes within B cells and plasma cells. While both cell types express antibody‐related genes, plasma cells generally exhibit significantly higher expression levels compared to B cells, with the exception of IGHE and IGHD, which show lower expression levels in plasma cells (Figure 2E). Subsequently, the TRUST4 algorithm was employed to generate BCR repertoire profiles for naive B cells, memory B cells, and plasma cells. There was no statistically significant difference observed in the abundance of B cells between the ACR and NR groups (Figure 2F). As for plasma cells, no comparison was made with the NR group cells due to their absence in NR. However, it is striking that the absolute abundance of BCR in plasma cells greatly surpassed that of B cells (~182‐fold) (Figure 2G). Consequently, our focus shifted to the BCR within plasma cells. It was discovered that heavy chains constituted 11% of the total weight and IGHG represented the largest proportion at 68%. IGHG1 accounted for 91.9% of the total weight within IGHG (Figure 2H). In conclusion, these findings highlight the critical role of IGHG, particularly IGHG1, in the BCR of TCMR. Furthermore, an analysis of immunoglobulin heavy chain variable region (IGHV) genes in the TCMR group was conducted using both single‐cell and bulk RNA‐seq datasets. Following the exclusion of low‐expression IGHV genes, six and three predominant IGHV genes were identified in single‐cell and bulk RNA‐seq datasets, respectively (Figure 2I,J). Notably, IGHV3‐23, IGHV3‐30, and IGHV4‐59 were consistently identified as top genes in both datasets, with significantly higher expression levels of IGHV3‐23 and IGHV3‐30 observed in the TCMR group compared to the STA group (Figure 2K).

### 3.3 Plasma Cell Infiltration Is Enhanced in TCMR and Is Associated With Poor Prognosis

**FIGURE 3:**
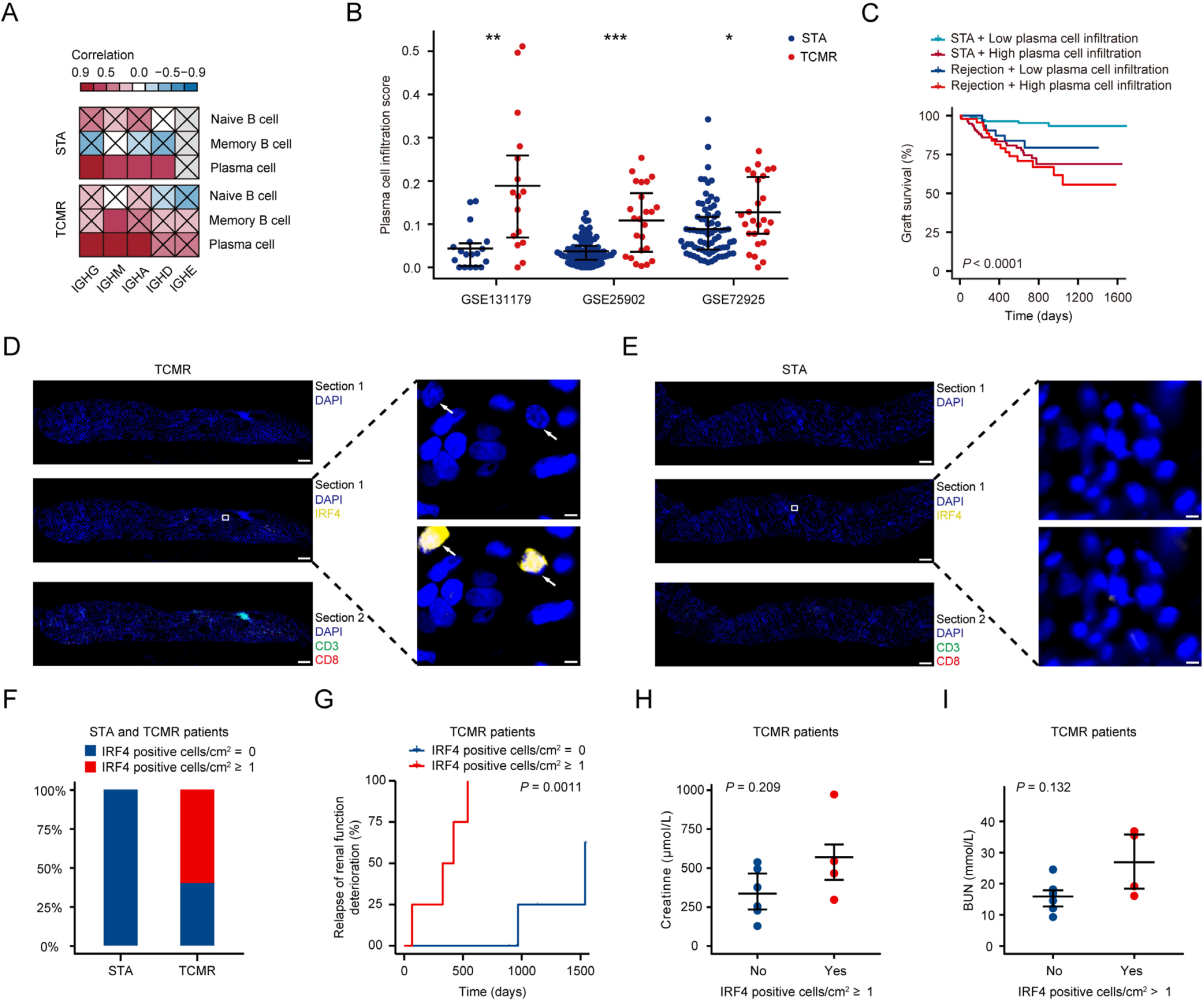
Exploration and validation of the role of plasma cells in different datasets. (A) Heatmap displays the correlation between the expression of estimated immune cell infiltration scores including Naive B and Memory B cells, and Plasma cells and B cell receptor‐related genes expression including IGHG, IGHM, IGHA, IGHD, and IGHE. (B) Beeswarm plots demonstrate the comparison of the Plasma cell infiltration score between the T cell‐mediated rejection (TCMR) group and the stable renal function (STA) group in three datasets. (C) K‐M survival curves show high and low Plasma cell infiltration between normal and rejection groups in GSE21374. Representative IF staining images of IRF4, CD3, and CD8 for the kidney biopsies with TCMR (D) and STA (E) samples in our hospital cohort. (F) Bar graph shows the ratio of IRF4 positive cells/cm^2^ = 0 and IRF4 positive cells/cm^2^ ≥ 1 between the TCMR group and the STA group in our hospital cohort. The K‐M curve represents relapse of renal function deterioration between IRF4 positive cells/cm^2^ = 0 and IRF4 positive cells/cm^2^ ≥ 1 in the TCMR group patients in our hospital cohort. Beeswarm plots demonstrate the comparison of creatinine and blood urea nitrogen (BUN) between the TCMR group and the STA group patients in our hospital cohort. * *p* < 0.05; **p < 0.01; ***p < 0.001. Data are presented as mean ± SEM.

Given the notably higher presence of plasma cells in the single‐cell RNA‐seq data, we proceeded to investigate the relationship between estimated immune cell infiltration and the abundance of each heavy chain subgroup in the bulk dataset (Figure 3A) and Table S3,S4. Consistently, plasma cells have a strong positive correlation with heavy chain subgroups, including IGHG, IGHA, and IGHM in the TCMR group (Figure 3A). Directly following this, we explored the extent of plasma cell infiltration in tissues using five independent TCMR datasets (Figure 3B). The plasma cell infiltration score in the TCMR group was significantly higher than that of the STA group in GSE131179 (*p* < 0.01), GSE25902 (*p* < 0.001), GSE72925 (*p* < 0.05), and the expression of plasma cell‐associated marker genes was significant in all three cohorts (Figure S4A–C). Moreover, an analysis of 282 kidney transplant biopsy specimens revealed a correlation between elevated plasma cell levels and diminished transplant survival rates in both normal and rejection transplant recipients (*p* < 0.0001, Figure 3C). To confirm these findings, histological examination through immunofluorescence (IF) was conducted on kidney biopsies from transplant patients at our hospital diagnosed with TCMR or STA, using IRF4 (also called multiple myeloma oncogene 1, MUM1) as a specific marker for plasma cells in human kidney [32]. Our study identified infiltration of plasma cells in TCMR, while absent in STA (Figure 3D,E). Among the patient cohort, 60% displayed notable plasma cell infiltration in the graft, contrasting starkly with the absence of such infiltration in STA cases (Figure 3F). Subsequent analysis of patient outcomes revealed a marked association between plasma cell tissue infiltration in TCMR cases and a heightened risk of transplant kidney function decline and recurrence (Figure 3G *p* = 0.0011). However, in terms of creatinine and blood urea nitrogen levels, which are indicators of graft kidney function, plasma cell tissue infiltration did not differ in TCMR patients (Figure 3H,I).

### 3.4 *MEI1* Is Identified as a Key BCR‐Related Gene in TCMR After Kidney Transplantation

**FIGURE 4:**
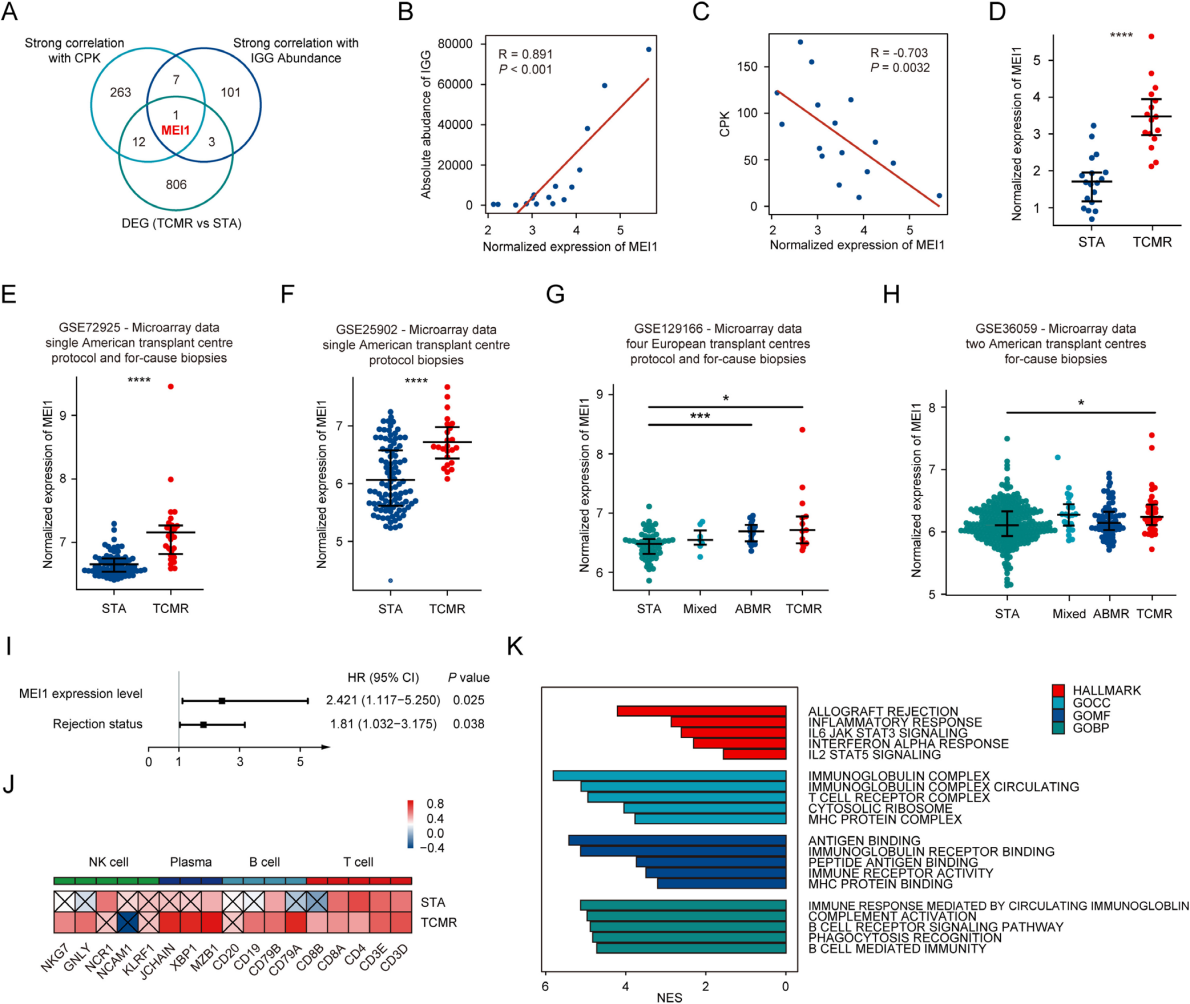
Exploration and validation of roles of *MEI1* in TCMR. (A) Venn plot shows genes with a strong correlation with CPK, a strong correlation with IGG Abundance, and differential expression genes. Scatter charts show the correlation of *MEI1* with absolute abundance (B) and CPK (C). Beeswam plots compare the expression of *MEI1* between the T cell‐mediated rejection (TCMR) group and the stable renal function (STA) group in different datasets including GSE131179 (D), GSE72925 (E), GSE25902 (F). Beeswam plots compare the expression of *MEI1* among STA, mix‐rejection, antibody‐mediated rejection, and TCMR groups in different datasets including GSE129166 (G) and GSE36059 (H). (I) Forest plot demonstrates graft survival for *MEI1* expression adjusted by rejection status in GSE21374. (J) Heatmap displays the correlation between the expression of *MEI1* and marker genes expression including NK cell, Plasma cell, B cell, and T cell in GSE131179. (K) Single‐gene GSEA of *MEI1* in GSE131179. * *p* < 0.05; ***p < 0.001; ****p < 0.0001. Data are presented as mean ± SEM.

Considering the significant increase in the absolute abundance and diversity of IGHG in the BCR of the TCMR group, we integrated IGHG CPK‐related genes, IGHG abundance‐related genes, and differentially expressed genes between the TCMR and the STA group to identify the crucial gene for BCR‐related immunity regulation (Figure 4A) and Table S5–7. Consequently, *MEI1* emerged as a novel key gene in TCMR, exhibiting a robust positive correlation with IGHG abundance (*R* = 0.891, Figure 4B), a strong negative correlation with IGHG CPK (lower CPK indicates clonal expansion; R = −0.703, Figure 4C), and a significantly increased gene expression level (*p* < 0.0001, Figure 4D). Then, four additional TCMR biopsy datasets were analyzed, namely GSE72925 (*p* < 0.0001, Figure 4E), GSE25902 (*p* < 0.0001, Figure 4F), GSE129166 (*p* < 0.05, Figure 4G), and GSE36059 (*p* < 0.05, Figure 4H), all indicating a significant upregulation of *MEI1* expression in the TCMR group compared to STA. While no significant difference in *MEI1* expression levels was observed between antibody‐mediated rejection (ABMR) and TCMR in GSE219166 and GSE36059 (Figure 4G,H). Furthermore, in the plasma cell dataset subdivided by surface heavy chain immunoglobulin, *MEI1* expression level was significantly higher in IgG classification than IgA and IgM in plasma cells, while there was no significant difference in circulating plasma cells Figure S4D. Notably, in the biopsy cohort GSE21374, multivariate Cox regression analysis revealed that *MEI1* expression, after adjusted for rejection status, remained a significant risk factor for graft survival in transplant recipients (HR = 2.421, *p* = 0.0251, Figure 4I). The KM curve indicated no significant difference between the two groups when stratifying *MEI1* expression in 1:1 and 2:1 ratio Figure S5A,B, but demonstrated distinctions between high and low *MEI1* expression groups when stratified in ratios of 1:2, 1:3, 1:4, 2:1, 3:1, and 4:1 Figure S5C–G. Afterward, further investigation into the functional relevance of the *MEI1* gene was conducted at the transcriptomic level. Specifically, a positive correlation was observed between *MEI1* expression and immune cell‐related marker genes, particularly in the TCMR group and plasma cell‐related genes (Figure 4J). The gene set enrichment analysis (GSEA) analysis highlighted enrichment pathways including ALLOGRAFT REJECTION, IMMUNOGLOBULIN COMPLEX, ANTIGEN BINDING, and IMMUNE RESPONSE MEDIATED BY CIRCULATING IMMUNOGLOBULIN in allografts with elevated *MEI1* levels (Figure 4K).

**FIGURE 5:**
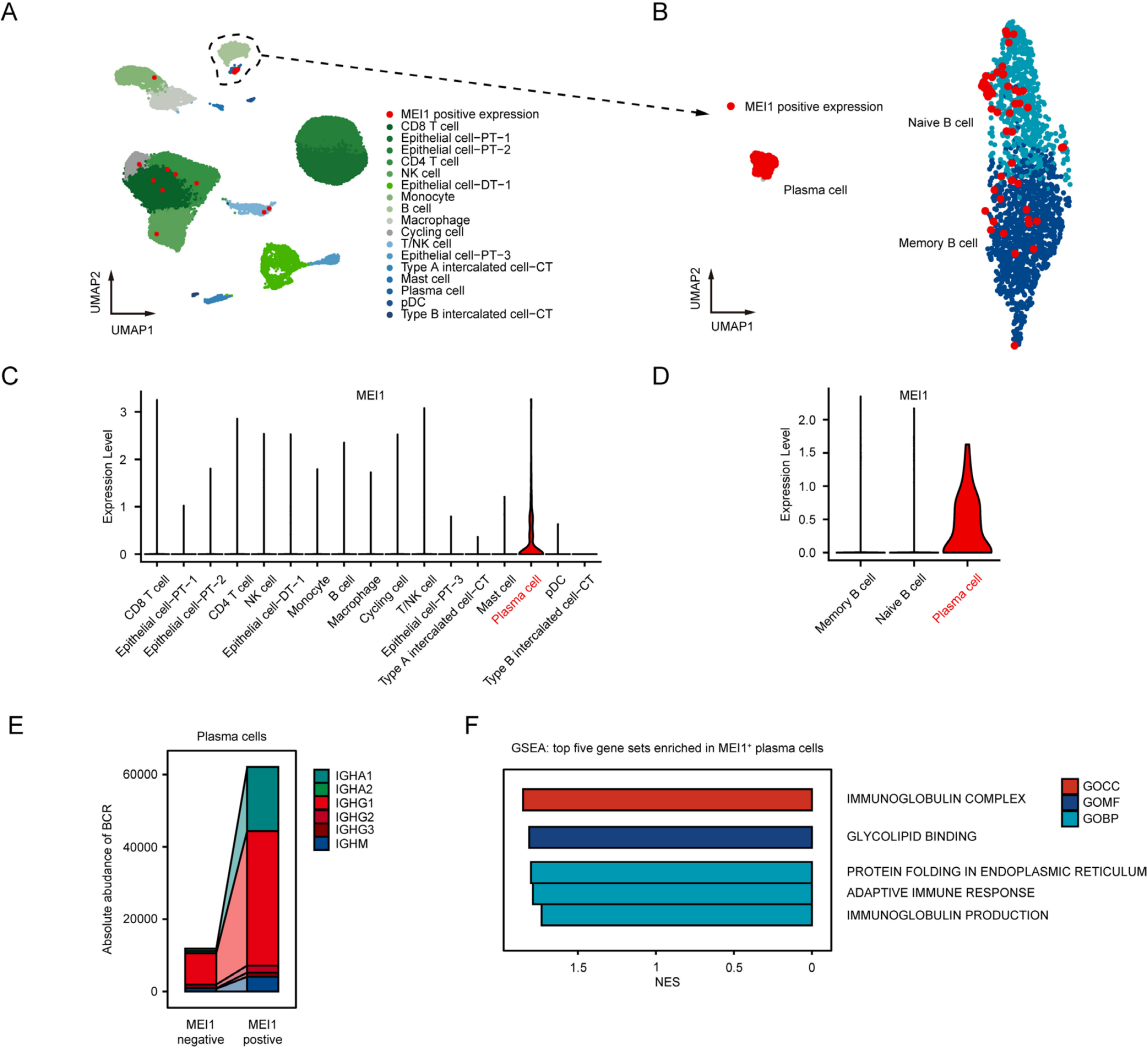
Single‐cell RNA‐seq data analysis of *MEI1*. (A) The uniform manifold approximation and projection (UMAP) plot from 15 acute cellular rejection biopsy samples and 6 non‐rejection biopsy samples in different cell types. Red dots represent cells that express ANXA2R. (B) UAMP plot of subtypes of Naive B, Memory B cells, and Plasma cells. Red dots represent cells that express *MEI1*. Violin plots show *MEI1* expression in different cell types (C) and subtypes of Naive B, Memory B cells, and Plasma cells (D). (E) Alluvial diagrams represent the difference expression of *MEI1* between the *MEI1* negative group and the *MEI1* positive group for absolute abundance. (F) Gene set enrichment analysis results of top five gene sets enriched in MEl1 positive plasma cells.

Subsequently, we investigated the expression level of *MEI1* at the single‐cell level. Utilizing single‐cell RNA‐seq data obtained from the Kidney Tissue Atlas, encompassing 20 healthy, 12 acute kidney injury, and 15 chronic kidney disease samples, the findings indicate a specific expression of *MEI1* in plasma cells across all cell populations Table S8. Subsequently, the dataset PRJNA974568 was utilized for additional investigation, revealing that *MEI1* displays the highest expression levels in plasma cells (Figure 5A–D). Consequently, plasma cells were selected for BCR repertoire analysis. In plasma cells, *MEI1* ^+^ cells displayed significantly higher absolute abundance compared to *MEI1* ^−^ cells, particularly in IGHG1 (Figure 5E). The GSEA result of *MEI1* ^+^ cells demonstrated that *MEI1* was significantly positively related to IMMUNOGLOBULIN COMPLEX, ADAPTIVE IMMUNE RESPONSE, and IMMUNOGLOBULIN PRODUCTION (Figure 5F).

### 3.5 *MEI1* Affects Antibody Secretion

**FIGURE 6:**
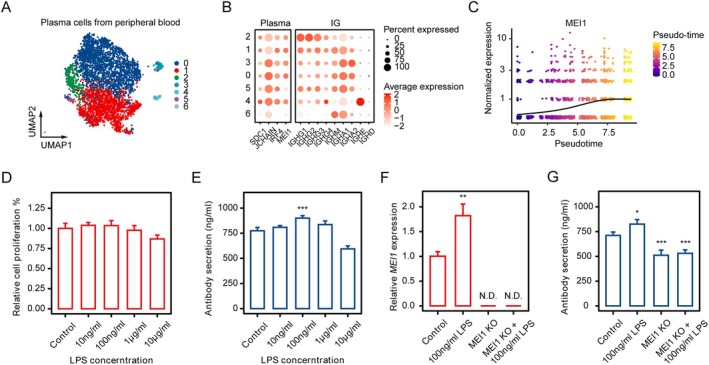
Analysis of *MEI1* affecting antibody secretion. (A) The uniform manifold approximation and projection (UMAP) plot from Plasma cells from three healthy adults in different subpopulations. (B) Dim plots demonstrate gene expression in different Plasma cell subpopulations. (C) Pseudotime analysis displays *MEI1* expression in different development times of cells. (D) Relative cell proliferation (%) in response to varying concentrations of LPS (0, 10 ng/mL, 100 ng/mL, 1 μg/mL, 10 μg/mL). (E) Antibody secretion (ng/ml) in response to varying concentrations of LPS (0, 10 ng/mL, 100 ng/mL, 1 μg/mL, 10 μg/mL). (F) Relative *MEI1* expression in response to LPS and *MEI1* knockout (KO) cells. Cells were treated with 100 ng/mL LPS for 48 h. *MEI1* KO cells and *MEI1* KO cells treated with 100 ng/mL LPS were both analyzed. (G) Antibody secretion (ng/ml) in response to 100 ng/mL LPS in wild‐type (WT) and *MEI1* KO cells. Cells were treated with different concentrations of LPS for 48 h. N.D. indicates not detectable. * *p* < 0.05; **p < 0.01; ***p < 0.001. Data are presented as mean ± SEM.

We further analyzed the single‐cell transcriptome of flow‐sorted (enabling enrichment of plasma cells for single‐cell RNA‐seq rather than only a small fraction of total cells) plasma cells (4088 cells) from three healthy adults (Figure 6A,B). Pseudo‐time analysis revealed that along with plasma cell maturation, *MEI1* expression levels gradually increased (Figure 6C). The human plasma cell leukemic cell line LP‐1 serves as a good cell model for investigating plasma cell maturation [33]. The dose of 100 ng/mL LPS for 48 h was found to be able to stimulate increased antibody secretion (Figure 6D,E). As shown in Figure S6A,B, MEI1 knockout did not reveal any significant difference in either cell viability or cell proliferation. Meanwhile, the expression level of *MEI1* was significantly up‐regulated (Figure 6F). Knockout of *MEI1* significantly impaired the antibody secretion and the effect of LPS on it (Figure 6G). Collectively, these findings suggest that MEI1 may modulate antibody secretion by participating in LPS‐mediated signaling pathways, potentially linking plasma cell activation with immune responses.

## 4 Discussion

Despite advances in immunosuppression, TCMR is still a severe unsolved clinical concern in transplantation, especially lacking targeted therapeutic strategies. In this study, we for the first time elucidated the landscape of BCR repertoire in human renal allografts with TCMR and STA at both bulk and single‐cell levels. BCR was found to be markedly expanded in TCMR, characterized by increased abundance and diversity of IgG. Infiltrated plasma cells were found to be the main holders of the expanded BCR, and its high infiltration level indicated a worse prognosis. Finally, *MEI1* was identified as a novel key BCR‐related gene in TCMR. Notably, the above results were consistently observed across multiple cohorts. Despite high heterogeneity among datasets, we still observed highly consistent BCR repertoire patterns and key gene expression profiles in TCMR, providing strong evidence for the robustness and reliability of our findings. Collectively, these data from multi‐omics revealed the underestimated roles of BCR and plasma cells in TCMR, offering new potential therapeutic targets.

In our data, the BCR repertoire was potently expanded in TCMR (~38 fold) compared to STA, indicating a non‐negligible involvement of BCR and B cell lineage in TCMR. Among all BCRs, IgG1 was found to have the top abundance elevation in TCMR (~51 fold) compared to STA and accounts for more than half of the total BCRs. IgG is the dominant immunoglobulin and can be divided into 4 distinct subclasses (IgG1 ~ 4) [34]. With the help of T cells, protein antigens usually trigger B cell class switching to IgG1 or IgG3, possessing distinct effector functions including phagocytosis, degranulation, antibody‐dependent cell‐mediated cytotoxicity (ADCC), complement activation, and agonistic signaling [34, 35]. Song et al. investigated the abundance and diversity of IGH in various tumor immunotherapy cohorts and found that the BCR repertoire could aid in predicting immune responses [36]. Higher levels of IgG1 + IgG3 are associated with stronger ADCC, antibody‐dependent cellular phagocytosis, and more crucially, better tumor immunotherapy outcomes. Different from cancer, higher expansion of BCRs and level of local B cells or plasma cells are mainly foes against allografts. Bharadwaj et al. reported that enrichment of serum IgG1 antibodies was widely associated with potentiated ADCC and contributed to graft rejection [37]. Taken together, our data show that IgG1 is the main participant in TCMR and, hence is the primary target for intervention. Interestingly, in our data, IGHV3‐23 consistently shows in the top abundant IGHV gene list in TCMR in both bulk and single‐cell data, echoing its potential association with rejection as previously reported. IGHV3‐23 is an IGHV gene and is initially investigated in B cell neoplasms such as chronic lymphocytic leukemia [38]. In the field of transplantation, Cheng et al. reported that the IGHV3‐23 gene dominated 61% of the 41 clones in 10 kidney transplant recipients [39]. Pineda et al. showed a significant increase of IGHV3‐23 in patients with multiple rejection episodes, suggesting its potential driving impact on rejection [40]. Consequently, the roles of IGHV3‐23 in reflecting the immune status and serving as an intervention target are worth further investigation in kidney transplantation.

According to single‐cell analysis, the expanded BCRs in TCMR were found to be predominantly expressed in plasma cells compared to naive B and memory B cells (~184 fold in total abundance), despite that the number of B cells is more than ten‐fold that of plasma cells. Not surprisingly, plasma cell infiltration levels are found to be significantly higher in TCMR in multiple independent transplant cohorts, which is further verified by IF staining of biopsies from our cohort. Speaking of plasma cell infiltration, there is a rare rejection type named plasma cell‐rich acute rejection (PCAR), which is defined by the heavy infiltration of mature plasma cells comprising over 10% of immune cells in the renal graft [41]. PCAR has worse graft survival and poses challenges in treatment, while its incidence rate is only approximately 1% in rejection [42]. Different from PCAR, our findings reveal a phenomenon that plasma cell infiltration is broadly enhanced in most TCMR samples across multiple independent cohorts with levels far from the diagnostic criteria of PCAR. Apart from heavy infiltrations in rare cases, plasma cells are involved in common cases of TCMR.

Prior observational studies reported the connection between plasma cell infiltration and drug hypersensitivity, infections, or post‐transplant lymphoproliferative disorders [42]. Infiltrated plasma cells have the propensity to aggregate within chronic inflammatory tissues, particularly in the context of autoimmune diseases such as systemic lupus erythematosus, autoimmune hemolytic anemia, and myasthenia gravis, where the secretion of autoantibodies by plasma cells plays the pivotal role in pathogenesis [43]. Evidence shows that plasma cells are specifically situated within the inflamed kidneys of murine models of lupus and patients with lupus erythematosus, thereby facilitating the localized synthesis of renal autoantibodies [44, 45]. In TCMR, the role of infiltrated plasma cells is poorly investigated. In our research, these infiltrated plasma cells in TCMR have potent BCR clonal amplification, especially IgG1, indicating an activated immune status. More importantly, we found that plasma cell infiltration is associated with a worse prognosis in recipients with TCMR or all recipients. Unfortunately, the specific mechanism of how these infiltrated plasma cells in TCMR contribute to a worse prognosis is unclear and worth future investigation. In addition, a recent meta‐analysis indicated that over one‐third of cases of TCMR would persist or recur following 2–9 months of standardized treatment, significantly leading to elevated risk of chronic graft injury and graft loss. Considering that current treatments of TCMR do not target mature plasma cells, the infiltrated plasma cells and expanded BCR may serve as novel potential targets for therapeutic intervention in patients with TCMR. Our previous research discovered the role of TCR repertoire in TCMR. Here, we extend those findings by demonstrating that T‐cell infiltration is accompanied by significant BCR repertoire remodeling and active plasma cell infiltration. The presence of this ‘concomitant’ humoral immunity provides a potential explanation for why some TCMR cases are refractory to standard T‐cell targeted treatments. Together, our works map a comprehensive immune receptor landscape, paving the way for dual‐targeting strategies.

Integrating BCR repertoire and multi‐omics data, and applying strict filter criteria, *MEI1* was identified as a novel key gene that was upregulated in TCMR with strong positive correlations with the abundance and diversity of IGHG. *MEI1*, originally identified as a meiotic‐programmed DNA double‐strand break‐forming protein, played a crucial role in meiotic recombination and the maintenance of normal meiotic processes. Previous research on *MEI1* primarily focused on meiosis‐induced sterility [46, 47, 48]. This is the first study to uncover its involvement in immunity. Our analysis of multiple cohorts revealed a notable upregulation of *MEI1* expression levels in TCMR, compared to those in STA. Single‐cell analysis revealed that *MEI1* is primarily expressed by plasma cells in renal allografts and its high expression was significantly associated with BCR expansion and immunoglobulin complex pathways in plasma cells. Moreover, according to pseudo‐time analysis, the expression level of *MEI1* was increased during plasma cell maturation. Consistently, our in vitro cell experimental results verified that *MEI1* was up‐regulated after stimulation of plasma cells and its knockout inhibited the ability of antibody secretion. More importantly, *MEI1* was identified as a significant independent risk factor for allograft survival after adjusting the rejection history. Taken together, *MEI1* could serve as a new potential therapeutic target and biomarker in TCMR and other diseases involving plasma cells or antibodies. However, it is necessary to consider that *MEI1* is essential for sperm division, making it need to be cautiously targeted for male transplant recipients with future fertility plans. Nevertheless, the high specificity of expression largely reduces the theoretical side effects of *MEI1*‐targeted therapy. It is worth mentioning that *MEI1* is only an example. On top of that, there are more potential key genes and mechanisms to be uncovered through resolving BCR repertoire in allografts with TCMR.

## 5 Conclusions

In conclusion, this study, for the first time, elucidated the landscape of BCR repertoire in renal allografts with TCMR through comprehensive analyses of multi‐omics data from ten datasets and in vitro experiments. Our findings highlight the importance of previously neglected BCR repertoire and allograft‐infiltrated plasma cells in TCMR, offering novel biomarkers and therapeutic targets.

## Author Contributions

He Zhang, Di Zhang, and Xiaopeng Hu initiated and designed the study. He Zhang, Di Zhang, Yuhong Hu, and Hao Zhang performed bioinformatics and statistical analyses, generated figures, and drafted the manuscript. Di Zhang and Zijian Zhang conducted the experimental work. Zijian Zhang and Xiaopeng Hu critically reviewed and revised the manuscript. All authors have read and approved the final version of the manuscript.

## Funding

This work was supported by the General Program of the National Natural Science Foundation of China grant 82 170 766 and 82 470 777 (Xiaopeng Hu) and the General Program of the Natural Science Foundation of Beijing grant 7 242 056 (Zijian Zhang).

## Disclosure

The authors have nothing to report.

## Ethics Statement

The study was conducted according to the guidelines of the Declaration of Helsinki and approved by the ethics committees of Beijing Chaoyang Hospital (Approval number 2023‐科‐16).

## Conflicts of Interest

The authors declare no conflicts of interest.

## Supporting information


**Data S1:** Supporting Information.

## Data Availability

All data generated or analyzed during this study were included in this article's methods section. Other data supporting this study's findings are available from the corresponding author upon reasonable request.

## References

[1] B. J. Nankivell , R. J. Borrows , C. L. S. Fung , P. J. O'Connell , R. D. M. Allen , and J. R. Chapman , “Natural History, Risk Factors, and Impact of Subclinical Rejection in Kidney Transplantation,” Transplantation 78, no. 2 (2004): 242, 10.1097/01.TP.0000128167.60172.CC. 15280685

[2] M. E. Seifert , M. V. Yanik , D. I. Feig , et al., “Subclinical Inflammation Phenotypes and Long‐Term Outcomes After Pediatric Kidney Transplantation,” American Journal of Transplantation 18, no. 9 (2018): 2189–2199, 10.1111/ajt.14933. 29766640 PMC6436389

[3] W. Hoffman , R. Mehta , D. R. Jorgensen , et al., “The Impact of Early Clinical and Subclinical T Cell–Mediated Rejection After Kidney Transplantation,” Transplantation 103, no. 7 (2019): 1457, 10.1097/TP.0000000000002560. 30747837

[4] P. Sood , W. S. Cherikh , A. E. Toll , R. B. Mehta , and S. Hariharan , “Kidney Allograft Rejection: Diagnosis and Treatment Practices in USA‐ A UNOS Survey,” Clinical Transplantation 35, no. 4 (2021): e14225, 10.1111/ctr.14225. 33455009

[5] J. Ho , G. N. Okoli , R. Rabbani , et al., “Effectiveness of T Cell‐Mediated Rejection Therapy: A Systematic Review and Meta‐Analysis,” American Journal of Transplantation 22, no. 3 (2022): 772–785, 10.1111/ajt.16907. 34860468 PMC9300092

[6] C. Rampersad , R. Balshaw , I. W. Gibson , et al., “The Negative Impact of T Cell–Mediated Rejection on Renal Allograft Survival in the Modern Era,” American Journal of Transplantation 22, no. 3 (2022): 761–771, 10.1111/ajt.16883. 34717048 PMC9299170

[7] T. Boehm , “Design Principles of Adaptive Immune Systems,” Nature Reviews. Immunology 11, no. 5 (2011): 307–317, 10.1038/nri2944. 21475308

[8] M. M Kenneth and W. Casey , Janeway's Immunobiology: Ninth International Student Edition (W.W. Norton & Company, 2016).

[9] M. G. Netea , A. Schlitzer , K. Placek , L. A. B. Joosten , and J. L. Schultze , “Innate and Adaptive Immune Memory: An Evolutionary Continuum in the Host's Response to Pathogens,” Cell Host & Microbe 25, no. 1 (2019): 13–26, 10.1016/j.chom.2018.12.006. 30629914

[10] K. Kiyotani , T. H. Mai , R. Yamaguchi , et al., “Characterization of the B‐Cell Receptor Repertoires in Peanut Allergic Subjects Undergoing Oral Immunotherapy,” Journal of Human Genetics 63, no. 2 (2018): 239–248, 10.1038/s10038-017-0364-0. 29192240

[11] D. M. Kurtz , M. R. Green , S. V. Bratman , et al., “Noninvasive Monitoring of Diffuse Large B‐Cell Lymphoma by Immunoglobulin High‐Throughput Sequencing,” Blood 125, no. 24 (2015): 3679–3687, 10.1182/blood-2015-03-635169. 25887775 PMC4463733

[12] S. Liu , X. L. Hou , W. G. Sui , Q. J. Lu , Y. L. Hu , and Y. Dai , “Direct Measurement of B‐Cell Receptor Repertoire's Composition and Variation in Systemic Lupus Erythematosus,” Genes and Immunity 18, no. 1 (2017): 22–27, 10.1038/gene.2016.45. 28053320

[13] F. Z. Cader , X. Hu , W. L. Goh , et al., “A Peripheral Immune Signature of Responsiveness to PD‐1 Blockade in Patients With Classical Hodgkin Lymphoma,” Nature Medicine 26, no. 9 (2020): 1468–1479, 10.1038/s41591-020-1006-1. PMC9673009 32778827

[14] D. Zhang , H. Zhang , J. Lu , and X. Hu , “Multiomics Data Reveal the Important Role of ANXA2R in T Cell‐Mediated Rejection After Renal Transplantation,” Transplantation 108, no. 2 (2024): 430–444, 10.1097/TP.0000000000004754. 37677931 PMC10798590

[15] V. N. Carpio , I. d. L. Noronha , H. L. Martins , et al., “Expression Patterns of B Cells in Acute Kidney Transplant Rejection,” Experimental and Clinical Transplantation 12, no. 5 (2014): 405–414. 25299368

[16] L. Schiffer , F. Wiehler , J. H. Bräsen , et al., “Chemokine CXCL13 as a New Systemic Biomarker for B‐Cell Involvement in Acute T Cell‐Mediated Kidney Allograft Rejection,” International Journal of Molecular Sciences 20, no. 10 (2019): 2552, 10.3390/ijms20102552. 31137652 PMC6567305

[17] A. Verma , T. Muthukumar , H. Yang , et al., “Urinary Cell Transcriptomics and Acute Rejection in Human Kidney Allografts,” JCI Insight 5, no. 4 (2020): e131552, 10.1172/jci.insight.131552. 32102984 PMC7101135

[18] Y. Shah , H. Yang , F. B. Mueller , et al., “Transcriptomic Signatures of Chronic Active Antibody‐Mediated Rejection Deciphered by RNA Sequencing of Human Kidney Allografts,” Kidney International 105, no. 2 (2024): 347–363, 10.1016/j.kint.2023.11.012. 38040290 PMC10841597

[19] P. F. Halloran , J. Chang , K. Famulski , et al., “Disappearance of T Cell‐Mediated Rejection Despite Continued Antibody‐Mediated Rejection in Late Kidney Transplant Recipients,” Journal of the American Society of Nephrology 26, no. 7 (2015): 1711–1720, 10.1681/ASN.2014060588. 25377077 PMC4483591

[20] A. Loupy , C. Lefaucheur , D. Vernerey , et al., “Molecular Microscope Strategy to Improve Risk Stratification in Early Antibody‐Mediated Kidney Allograft Rejection,” Journal of the American Society of Nephrology 25, no. 10 (2014): 2267–2277, 10.1681/ASN.2013111149. 24700874 PMC4178445

[21] E. Van Loon , S. Gazut , S. Yazdani , et al., “Development and Validation of a Peripheral Blood mRNA Assay for the Assessment of Antibody‐Mediated Kidney Allograft Rejection: A Multicentre, Prospective Study,” eBioMedicine 46 (2019): 463–472, 10.1016/j.ebiom.2019.07.028. 31378695 PMC6710906

[22] T. Sigdel , M. Nguyen , J. Liberto , et al., “Assessment of 19 Genes and Validation of CRM Gene Panel for Quantitative Transcriptional Analysis of Molecular Rejection and Inflammation in Archival Kidney Transplant Biopsies,” Front Med (Lausanne) 6 (2019): 213, 10.3389/fmed.2019.00213. 31632976 PMC6781675

[23] T. K. Sigdel , O. Bestard , N. Salomonis , et al., “Intragraft Antiviral‐Specific Gene Expression as a Distinctive Transcriptional Signature for Studies in Polyomavirus‐Associated Nephropathy,” Transplantation 100, no. 10 (2016): 2062–2070, 10.1097/TP.0000000000001214. 27140517 PMC5235336

[24] M. Naesens , P. Khatri , L. Li , et al., “Progressive Histological Damage in Renal Allografts Is Associated With Expression of Innate and Adaptive Immunity Genes,” Kidney International 80, no. 12 (2011): 1364–1376, 10.1038/ki.2011.245. 21881554 PMC4492284

[25] G. Einecke , J. Reeve , B. Sis , et al., “A Molecular Classifier for Predicting Future Graft Loss in Late Kidney Transplant Biopsies,” Journal of Clinical Investigation 120, no. 6 (2010): 1862–1872, 10.1172/JCI41789. 20501945 PMC2877953

[26] T. Shi , A. R. Burg , J. T. Caldwell , et al., “Single‐Cell Transcriptomic Analysis of Renal Allograft Rejection Reveals Insights Into Intragraft TCR Clonality,” Journal of Clinical Investigation 133, no. 14 (2023): 191, 10.1172/JCI170191. PMC10348771 37227784

[27] D. Alameda , I. Goicoechea , M. Vicari , et al., “Tumor Cells in Light‐Chain Amyloidosis and Myeloma Show Distinct Transcriptional Rewiring of Normal Plasma Cell Development,” Blood 138, no. 17 (2021): 1583–1589, 10.1182/blood.2020009754. 34133718

[28] L. Song , D. Cohen , Z. Ouyang , Y. Cao , X. Hu , and X. S. Liu , “TRUST4: Immune Repertoire Reconstruction From Bulk and Single‐Cell RNA‐Seq Data,” Nature Methods 18, no. 6 (2021): 627–630, 10.1038/s41592-021-01142-2. 33986545 PMC9328942

[29] B. Chen , M. S. Khodadoust , C. L. Liu , A. M. Newman , and A. A. Alizadeh , “Profiling Tumor Infiltrating Immune Cells With CIBERSORT,” Methods in Molecular Biology 1711 (2018): 243–259, 10.1007/978-1-4939-7493-1_12. 29344893 PMC5895181

[30] A. Subramanian , P. Tamayo , V. K. Mootha , et al., “Gene Set Enrichment Analysis: A Knowledge‐Based Approach for Interpreting Genome‐Wide Expression Profiles,” Proceedings of the National Academy of Sciences of the United States of America 102, no. 43 (2005): 15545–15550, 10.1073/pnas.0506580102. 16199517 PMC1239896

[31] X. Qiu , Q. Mao , Y. Tang , et al., “Reversed Graph Embedding Resolves Complex Single‐Cell Trajectories,” Nature Methods 14, no. 10 (2017): 979–982, 10.1038/nmeth.4402. 28825705 PMC5764547

[32] M. Meylan , F. Petitprez , E. Becht , et al., “Tertiary Lymphoid Structures Generate and Propagate Anti‐Tumor Antibody‐Producing Plasma Cells in Renal Cell Cancer,” Immunity 55, no. 3 (2022): 527–541, 10.1016/j.immuni.2022.02.001. 35231421

[33] L. Pegoraro , F. Malavasi , G. Bellone , et al., “The Human Myeloma Cell Line LP‐1: A Versatile Model in Which to Study Early Plasma‐Cell Differentiation and c‐Myc Activation,” Blood 73, no. 4 (1989): 1020–1027. 2784066

[34] N. M. Valenzuela and S. Schaub , “The Biology of IgG Subclasses and Their Clinical Relevance to Transplantation,” Transplantation 102, no. 1S Suppl 1 (2018): S7–S13, 10.1097/TP.0000000000001816. 29266057

[35] G. Vidarsson , G. Dekkers , and T. Rispens , “IgG Subclasses and Allotypes: From Structure to Effector Functions,” Frontiers in Immunology 5 (2014): 520, 10.3389/fimmu.2014.00520. 25368619 PMC4202688

[36] L. Song , Z. Ouyang , D. Cohen , et al., “Comprehensive Characterizations of Immune Receptor Repertoire in Tumors and Cancer Immunotherapy Studies,” Cancer Immunology Research 10, no. 7 (2022): 788–799, 10.1158/2326-6066.CIR-21-0965. 35605261 PMC9299271

[37] P. Bharadwaj , S. Shrestha , T. Pongracz , et al., “Afucosylation of HLA‐Specific IgG1 as a Potential Predictor of Antibody Pathogenicity in Kidney Transplantation,” Cell Reports Medicine 3, no. 11 (2022): 100818, 10.1016/j.xcrm.2022.100818. 36384101 PMC9729883

[38] R. Bomben , M. Dal‐Bo , D. Benedetti , et al., “Expression of Mutated IGHV3‐23 Genes in Chronic Lymphocytic Leukemia Identifies a Disease Subset With Peculiar Clinical and Biological Features,” Clinical Cancer Research 16, no. 2 (2010): 620–628, 10.1158/1078-0432.CCR-09-1638. 20068100

[39] J. Cheng , A. Torkamani , R. K. Grover , et al., “Ectopic B‐Cell Clusters That Infiltrate Transplanted Human Kidneys Are Clonal,” Proceedings of the National Academy of Sciences of the United States of America 108, no. 14 (2011): 5560–5565, 10.1073/pnas.1101148108. 21415369 PMC3078383

[40] S. Pineda , T. K. Sigdel , J. M. Liberto , F. Vincenti , M. Sirota , and M. M. Sarwal , “Characterizing Pre‐Transplant and Post‐Transplant Kidney Rejection Risk by B Cell Immune Repertoire Sequencing,” Nature Communications 10, no. 1 (2019): 1906, 10.1038/s41467-019-09930-3. PMC6479061 31015506

[41] D. Desvaux , S. Le Gouvello , M. Pastural , et al., “Acute Renal Allograft Rejections With Major Interstitial Oedema and Plasma Cell‐Rich Infiltrates: High Gamma‐Interferon Expression and Poor Clinical Outcome,” Nephrology, Dialysis, Transplantation 19, no. 4 (2004): 933–939, 10.1093/ndt/gfh027. 15031352

[42] J. Hasegawa , K. Honda , K. Omoto , et al., “Clinical and Pathological Features of Plasma Cell‐Rich Acute Rejection After Kidney Transplantation,” Transplantation 102, no. 5 (2018): 853–859, 10.1097/TP.0000000000002041. 29319615

[43] P. D. Pioli , “Plasma Cells, the Next Generation: Beyond Antibody Secretion,” Frontiers in Immunology 10 (2019): 2768, 10.3389/fimmu.2019.02768. 31824518 PMC6883717

[44] T. Alexander , R. Sarfert , J. Klotsche , et al., “The Proteasome Inhibitior Bortezomib Depletes Plasma Cells and Ameliorates Clinical Manifestations of Refractory Systemic Lupus Erythematosus,” Annals of the Rheumatic Diseases 74, no. 7 (2015): 1474–1478, 10.1136/annrheumdis-2014-206016. 25710470 PMC4484251

[45] K. Hofmann , A. K. Clauder , and R. A. Manz , “Targeting B Cells and Plasma Cells in Autoimmune Diseases,” Frontiers in Immunology 9 (2018): 835, 10.3389/fimmu.2018.00835. 29740441 PMC5924791

[46] A. Nore , A. B. Juarez‐Martinez , J. Clément , et al., “TOPOVIBL‐REC114 Interaction Regulates Meiotic DNA Double‐Strand Breaks,” Nature Communications 13, no. 1 (2022): 7048, 10.1038/s41467-022-34799-0. PMC9671922 36396648

[47] J. Dong , H. Zhang , X. Mao , et al., “Novel Biallelic Mutations in MEI1: Expanding the Phenotypic Spectrum to Human Embryonic Arrest and Recurrent Implantation Failure,” Human Reproduction 36, no. 8 (2021): 2371–2381, 10.1093/humrep/deab118. 34037756

[48] N. M. P. Nguyen , Z. J. Ge , R. Reddy , et al., “Causative Mutations and Mechanism of Androgenetic Hydatidiform Moles,” American Journal of Human Genetics 103, no. 5 (2018): 740–751, 10.1016/j.ajhg.2018.10.007. 30388401 PMC6218808

