# Supplementary material for: Multi‐Omic Profiling of T Cell‐Mediated Rejection After Kidney Transplantation Reveals B Cell Receptor Repertoire Expansion and Its Prognostic Relevance
Source: FASEB J. 2025 Dec 30;40(1):e71407. doi: 10.1096/fj.202502448RR (PMC12752722; doi:10.1096/fj.202502448RR)
Supplement: Supplementary file 1 — Data S1: Supporting Information. [file FSB2-40-e71407-s001.pdf]

Supplementary Figures and Figure Legends

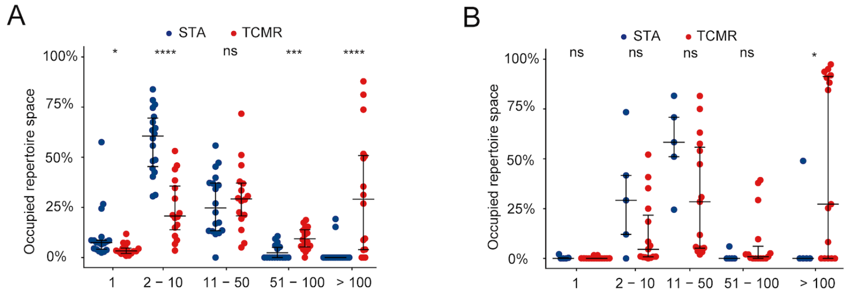

Supplementary Figure 1. Beeswarm plots demonstrate the comparison of clonality with specific counts between the T cell-mediated rejection (TCMR) group and the stable renal function (STA) group in bulk RNA seq (A) and single-cell RNA seq (B) datasets. * P < 0.05; *** P < 0.001; **** P < 0.0001. Data are presented as mean ± SEM.

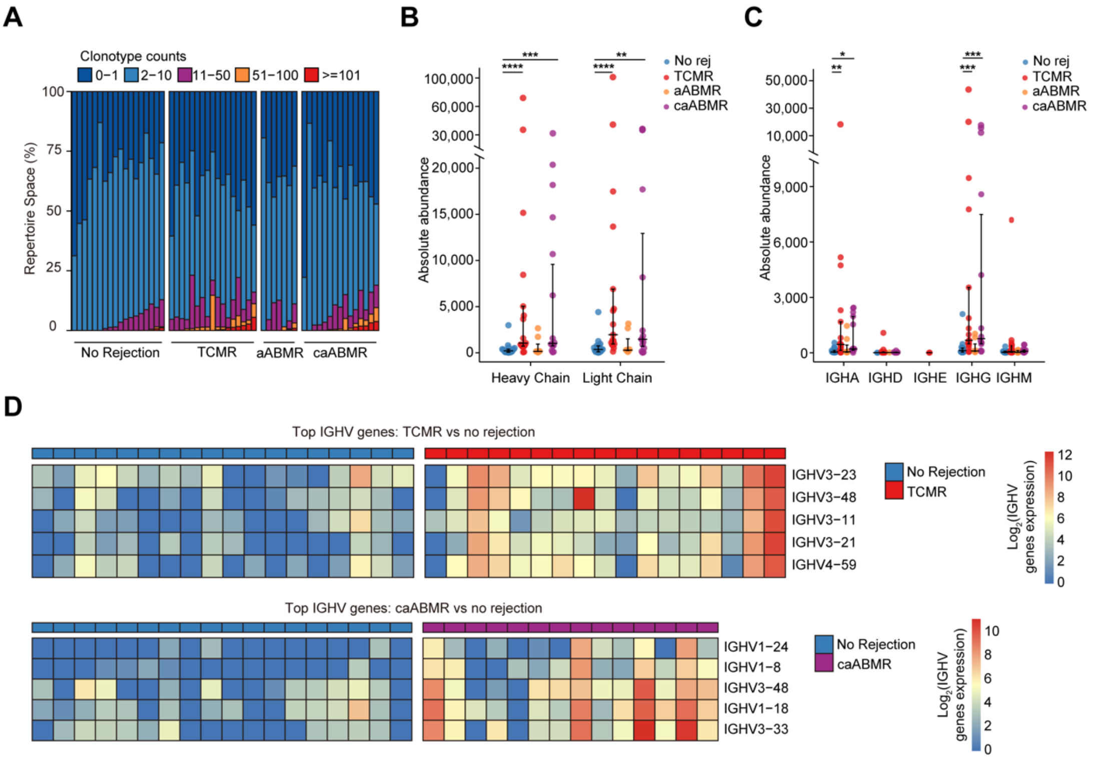

Supplementary Figure 2. Construction and analysis of B cell receptor repertoire in bulk dataset GSE232825. (A) The histogram plot shows the clonality of each sample with specific counts. Beeswarm plots demonstrate the comparison of the absolute abundance in heavy chain and light chain (B), heavy chain subdivision including IGHA, IGHD, IGHE, IGHG, IGHM (C). (D) The heatmap displays the $\log_2$-transformed expression levels of the top IGHV gene in each sample, identified from comparisons of TCMR versus non-rejection and caABMR versus non-rejection. * $P < 0.05$; ** $P < 0.01$; *** $P < 0.001$; **** $P < 0.0001$. Data are presented as mean ± SEM.

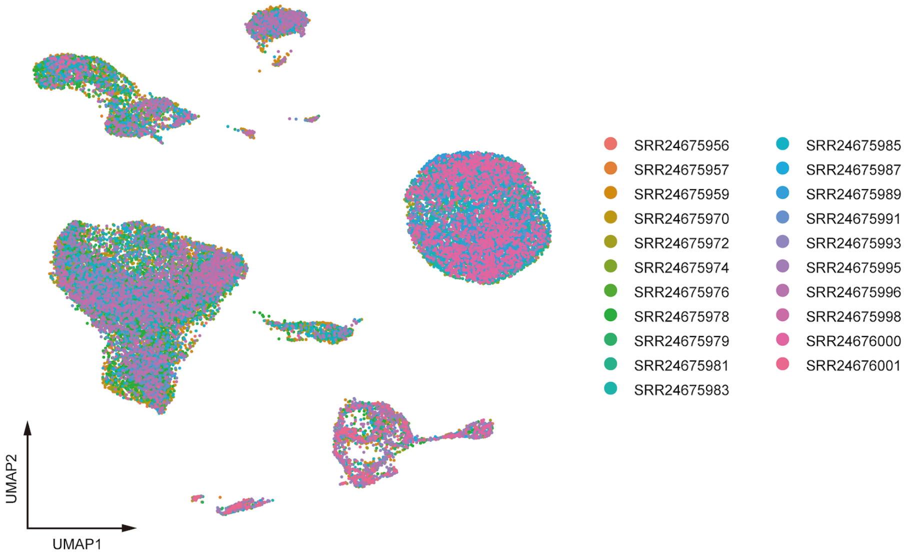

Supplementary Figure 3. UAMP plot from 15 acute cellular rejection biopsy samples and 6 non-rejection biopsy samples.

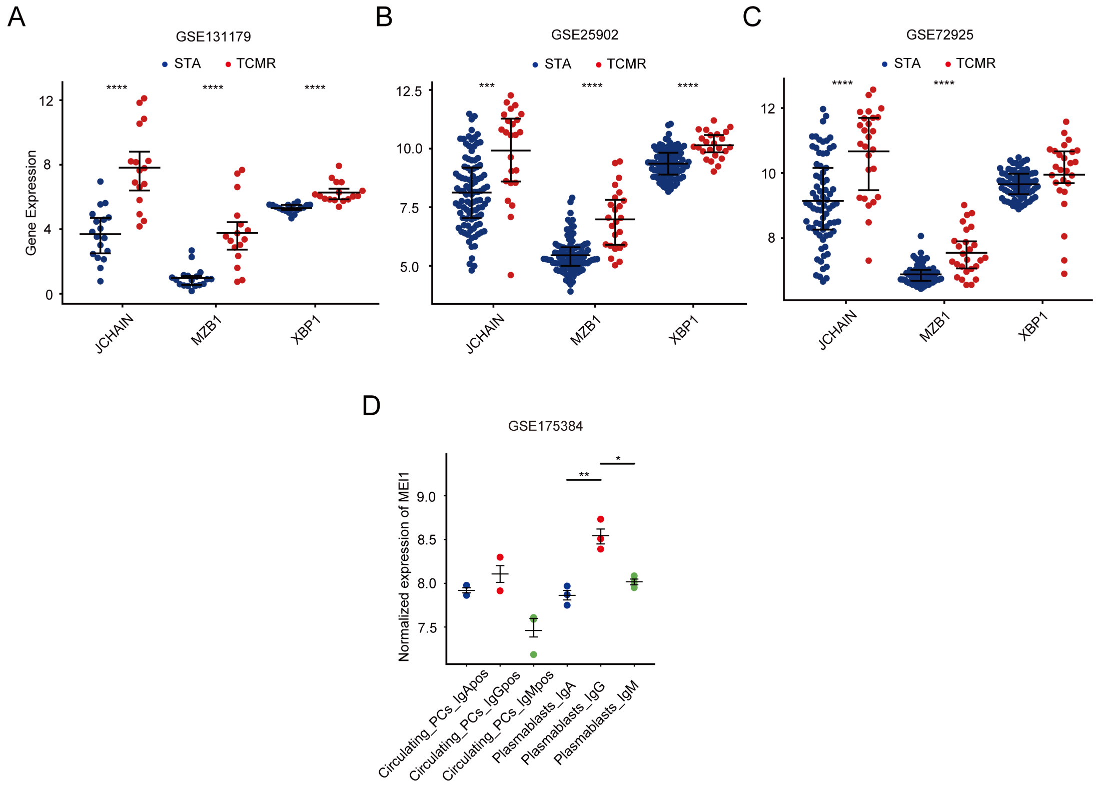

Supplementary Figure 4. Beeswarm plots show the comparison of plasma marker genes including JCHIAN, MZB1, and XBP1 between the T cell-mediated rejection (TCMR) group and the stable renal function (STA) group in GSE131179 (A), GSE25902 (B), and GSE72925 (C). (D) Beeswam plot compares the expression of *MEI1* between the TCMR and the STA group in different plasma cell subpopulations. * $P < 0.05$; ** $P < 0.01$; *** $P < 0.001$; **** $P < 0.0001$. Data are presented as mean ± SEM.

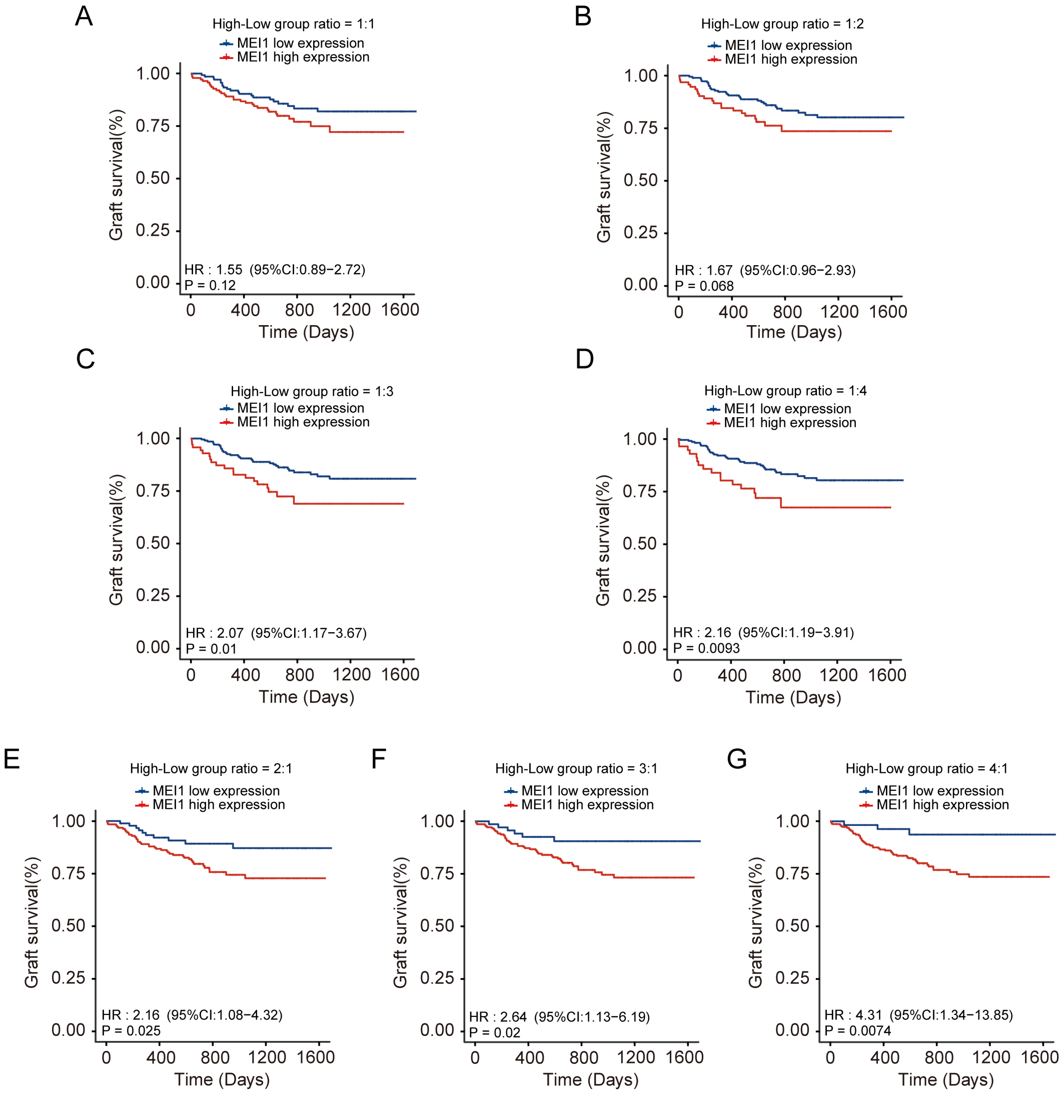

Supplementary Figure 5. The K-M curves display *MEI* expression stratified into 1:1 (A), 2:1 (B),1:2 (C), 1:3 (D), 1:4 (E), 2:1 (F), 3:1 (G), and 4:1 (H) between normal and rejection groups in GSE21374.

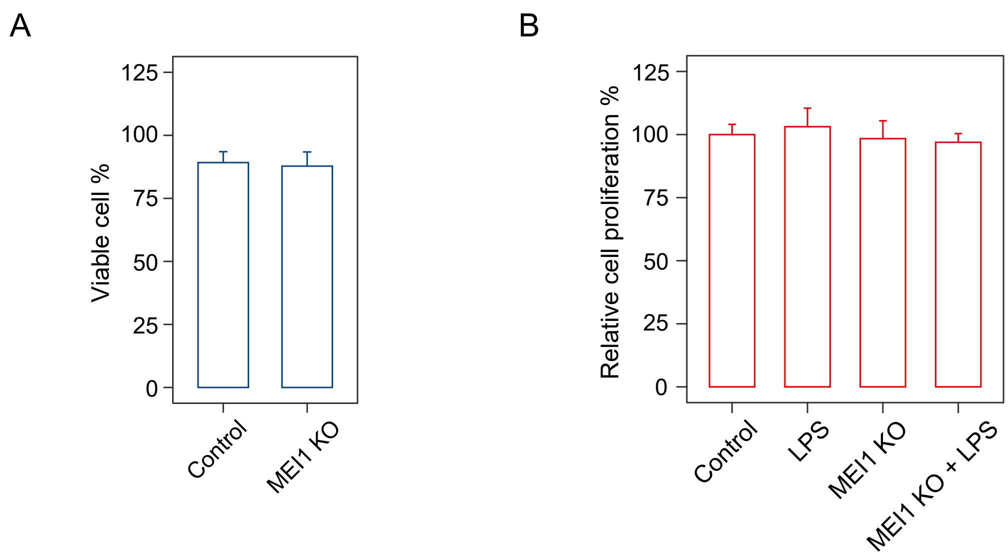

Supplementary Figure 6. Effects of MEI1 knockout and LPS stimulation on cell viability and proliferation. (A) Relative viable cell percentage in control and MEI1 knockout (MEI1 KO) cells. (B) Relative cell proliferation (%) in control, 100 ng/ml LPS-treated, MEI1 KO, and MEI1 KO cells treated with 100 ng/ml LPS. Cells were treated with LPS for 48 hours. Data are presented as mean ± SEM.

Supplementary Tables

Supplementary Table 1. Clinical characteristics of patients in this study.

ID	Group	Age	Gender	Creatinine (μmol/L)	BUN (mmol/L)	Follow-up time after biopsy	Type of donor	Induction regimen	Immuno-suppressive regimen
CYKT001	TCMR	29	Male	227.9	9.29	1539	DCD	rATG	Tac+MMF+PED
CYKT002	TCMR	36	Male	254.8	12.07	958	DCD	Bas	Tac+EC-MPS+PED
CYKT003	TCMR	54	Female	128.3	14.57	896	DCD	-	Tac+MMF+PED
CYKT004	TCMR	30	Female	297	16.08	420	DCD	rATG	Tac+MMF+PED
CYKT005	TCMR	26	Male	377	16.86	1547	DCD	rATG	Tac+MMF+MPED
CYKT006	TCMR	48	Male	495.1	18.19	1133	DCD	rATG	Tac+MMF+PED
CYKT007	TCMR	52	Male	544.6	19.19	66	DCD	-	CsA+MMF+PED
CYKT008	TCMR	33	Male	538.2	24.52	968	DCD	Bas	Tac+ Mizoribine +PED
CYKT009	TCMR	31	Female	466.9	35.45	540	DCD	rATG	Tac+MMF+PED
CYKT010	TCMR	38	Male	972.2	36.82	327	DCD	Bas	Tac+EC-MPS+PED
CYKT011	STA	20	Male	165.1	6.9	1511	DCD	-	CsA+MMF+PED
CYKT012	STA	55	Male	237.8	9.66	335	DCD	Bas	Tac+EC-MPS
CYKT013	STA	24	Male	552.1	9.93	1590	DCD	rATG	Tac+MMF+PED
CYKT014	STA	39	Male	505.8	10.25	1673	DCD	Bas	Tac+EC-MPS+PED
CYKT015	STA	45	Male	553.7	11.23	1606	DCD	rATG	Tac+EC-MPS+PED
CYKT016	STA	50	Male	314.7	13.59	1745	DCD	rATG	Tac+MMF+PED
CYKT017	STA	50	Male	545.3	15.83	351	DCD	rATG	Tac+MMF+PED
CYKT018	STA	52	Male	645.4	19.01	1181	DCD	rATG	Tac+MMF+PED
CYKT019	STA	47	Male	779.5	24.3	1609	DCD	rATG	Tac+EC-MPS+PED
CYKT020	STA	65	Male	474.9	30.1	130	DCD	rATG	Tac+MMF+PED

BUN: blood urea nitrogen; RRFD: Relapse of renal function deterioration; TCMR: T cell-mediated rejection; STA: stable renal function; DCD: donation after cardiac death; rATG: rabbit anti-human thymocyte immunoglobulin; Bas: Basiliximab; Tac: tacrolimus; MMF: mycophenolate mofetil; PED: prednisone; MPED: methylprednisone; CsA: Cyclosporine A; EC-MPS: enteric-coated mycophenolate sodium.

Supplementary Table 2. Information of all designed single-guide RNAs.

sequence	start	end	strand
TGTGAGGCAGGCGGCGACGG	5	34	+
GCCCAGGAGAGAGGAAGAGG	38	67	+
CGCAGGCCAGGCACAGGCGG	111	140	-
TGCGCACTAACGACACGCCG	153	182	-

Supplementary Table 3. Correlations between estimated immune cell infiltration and the abundance of each heavy chain subgroup in the STA group of GSE131179.

Immune cell	IGHG	IGHM	IGHA	IGHD	IGHE
B cell naive	0.438	0.133	0.404	0.07	NA
B cell memory	-0.355	0.049	-0.283	-0.341	NA
B cell plasma	0.887	0.664	0.612	0.519	NA
T cell CD8+	0.373	0.331	0.405	-0.006	NA
T cell CD4+ naive	0.683	0.287	0.686	0.036	NA
T cell CD4+ memory resting	-0.397	-0.059	-0.174	-0.17	NA
T cell CD4+ memory activated	NA	NA	NA	NA	NA
T cell follicular helper	-0.241	-0.043	-0.217	-0.096	NA
T cell regulatory (Tregs)	-0.235	-0.194	-0.368	-0.051	NA
T cell gamma delta	0.456	0.169	0.367	-0.198	NA
NK cell resting	-0.552	-0.385	-0.497	-0.061	NA
NK cell activated	0.379	0.207	0.457	0.196	NA
Monocyte	-0.22	-0.053	-0.012	-0.35	NA
Macrophage M0	0.033	0.154	0.116	0.33	NA
Macrophage M1	0.292	0.105	0.349	-0.165	NA
Macrophage M2	0.001	-0.079	-0.096	-0.45	NA
Myeloid dendritic cell resting	0.147	0.007	0.074	-0.275	NA
Myeloid dendritic cell activated	NA	NA	NA	NA	NA
Mast cell activated	0.088	0.006	-0.099	-0.005	NA
Mast cell resting	NA	NA	NA	NA	NA
Eosinophil	NA	NA	NA	NA	NA

Neutrophil	0.087	0.056	-0.195	0.193	NA

STA: stable renal function; IGHG, Immunoglobulin Heavy Constant Gamma; IGHM, Immunoglobulin Heavy Constant Mu; IGHA, Immunoglobulin Heavy Constant Alpha; IGHD, Immunoglobulin Heavy Constant Delta; IGHE, Immunoglobulin Heavy Constant Epsilon; NA, not available.

Supplementary Table 4. Correlations between estimated immune cell infiltration and the abundance of each heavy chain subgroup in the TCMR group of GSE131179.

Immune cell	IGHG	IGHM	IGHA	IGHD	IGHE
B cell naive	0.198	-0.007	0.15	-0.183	-0.365
B cell memory	0.213	0.508	0.388	0.246	0.144
B cell plasma	0.826	0.72	0.753	0.338	0.42
T cell CD8+	0.382	0.28	0.171	-0.084	-0.14
T cell CD4+ naive	-0.42	-0.42	-0.42	0.029	-0.067
T cell CD4+ memory resting	0.015	-0.102	-0.098	-0.472	0.254
T cell CD4+ memory activated	0.061	-0.122	0.129	0.331	-0.17
T cell follicular helper	0.106	-0.058	0.133	-0.027	-0.365
T cell regulatory (Tregs)	-0.29	-0.359	-0.259	0.13	0.196
T cell gamma delta	0.281	-0.077	0.009	-0.161	-0.086
NK cell resting	0.028	0.028	0.084	-0.314	-0.067
NK cell activated	0.035	0.205	0.183	0.586	0.197
Monocyte	0.096	0.046	-0.097	0.088	-0.028
Macrophage M0	-0.172	-0.11	0.102	0.287	-0.147
Macrophage M1	-0.009	-0.062	-0.015	0.108	0.084
Macrophage M2	-0.021	-0.178	-0.182	-0.305	-0.252
Myeloid dendritic cell resting	0.107	0.099	0.081	0.21	-0.028
Myeloid dendritic cell activated	NA	NA	NA	NA	NA
Mast cell activated	-0.335	-0.021	-0.132	0.53	-0.196
Mast cell resting	0.252	-0.056	0.196	-0.029	-0.067
Eosinophil	NA	NA	NA	NA	NA

Neutrophil	-0.364	-0.14	-0.308	0.143	-0.067

TCMR: T Cell-Mediated Rejection; IGHG, Immunoglobulin Heavy Constant Gamma; IGHM, Immunoglobulin Heavy Constant Mu; IGHA, Immunoglobulin Heavy Constant Alpha; IGHD, Immunoglobulin Heavy Constant Delta; IGHE, Immunoglobulin Heavy Constant Epsilon; NA, not available.

Supplementary Table 5. IGHG abundance-related genes in TCMR samples in GSE131179.

Gene	Correlation	*P* value
MZB1	0.959	<0.001
IGHV3-65	0.953	<0.001
IGHGP	0.947	<0.001
IGHG4	0.941	<0.001
IGHV3-22	0.935	<0.001
IGLC2	0.935	<0.001
ANKRD36BP2	0.932	<0.001
IGKC	0.932	<0.001
LINC02362	0.932	<0.001
IGLC3	0.929	<0.001
IGHV3-16	0.921	<0.001
IGHV3-33	0.921	<0.001
IGHV4-61	0.918	<0.001
IGHV3-21	0.915	<0.001
IGHV3-30	0.915	<0.001
IGHG1	0.912	<0.001
IGHG3	0.912	<0.001
IGLL5	0.912	<0.001
IGHV3-71	0.911	<0.001
FCRL5	0.909	<0.001
IGLV1-51	0.909	<0.001
IGHV3-11	0.907	<0.001
IGHV4-28	0.900	<0.001
IGKV3D-20	0.897	<0.001
FAM30A	0.897	<0.001
TNFRSF17	0.897	<0.001
IGLV3-19	0.895	<0.001
POU2AF1	0.894	<0.001
IGHM	0.894	<0.001
IGHV3-15	0.894	<0.001
IGHV3-43	0.892	<0.001
IGLV1-47	0.891	<0.001
IGLV2-23	0.891	<0.001
MEI1	0.891	<0.001
IGKV1-5	0.888	<0.001
IGHA1	0.888	<0.001
IGHV3-23	0.888	<0.001
IGKV1D-8	0.888	<0.001
AC106882.1	0.886	<0.001
CD79A	0.885	<0.001

IGLC1	0.885	<0.001
IGHV3-64	0.884	<0.001
MIXL1	0.884	<0.001
IGKV3-11	0.882	<0.001
IGKV3-20	0.882	<0.001
IGHV4-59	0.882	<0.001
IGHV3-13	0.876	<0.001
IGHV1-18	0.876	<0.001
IGHV3-66	0.876	<0.001
IGKV1-6	0.876	<0.001
IGHV3-48	0.874	<0.001
JCHAIN	0.871	<0.001
IGLC7	0.870	<0.001
IGKV1-39	0.868	<0.001
IGKV1D-39	0.868	<0.001
IGLV2-11	0.868	<0.001
IGHV4-55	0.864	<0.001
IGKV3D-15	0.859	<0.001
IGKV1-9	0.856	<0.001
IGHG2	0.856	<0.001
IGKV1D-12	0.853	<0.001
IGKV2D-28	0.850	<0.001
IGLV1-44	0.850	<0.001
AC034105.1	0.848	<0.001
IGHV4-31	0.848	<0.001
IGKV1-17	0.847	<0.001
IGHV3-41	0.844	<0.001
AC135068.2	0.844	<0.001
IGLV2-18	0.844	<0.001
DERL3	0.844	<0.001
IGHV3OR16-9	0.843	<0.001
IGKV3D-11	0.841	<0.001
IGHV5-51	0.841	<0.001
IGKV1-13	0.841	<0.001
IGHV3-49	0.839	<0.001
IGHA2	0.838	<0.001
IGKV2-28	0.832	<0.001
IGLV2-14	0.829	<0.001
IRF4	0.826	<0.001
IGHV3-53	0.826	<0.001
IGLV3-1	0.826	<0.001
IGHV4-4	0.824	<0.001
IGHV3OR16-8	0.822	<0.001

IGKV3OR2-268	0.821	<0.001
IGKV3-15	0.821	<0.001
IGKV3D-7	0.821	<0.001
IGHV3OR16-11	0.818	<0.001
TMEM130	0.815	<0.001
IGHV1-2	0.815	<0.001
LINC02576	0.801	<0.001
IGHV3-20	0.799	<0.001
BHLHA15	0.796	<0.001
IGKV1D-17	0.795	<0.001
AL365361.1	0.794	<0.001
IGHV3-74	0.794	<0.001
IGLV3-25	0.794	<0.001
IGLV3-21	0.794	<0.001
AC142381.1	0.794	<0.001
IGKV1-12	0.791	<0.001
IGKV3-7	0.788	<0.001
IGKV2D-30	0.785	<0.001
IGHV4-39	0.782	<0.001
AC135068.8	0.782	<0.001
IGHV1OR15-1	0.782	<0.001
IGHV1-12	0.782	<0.001
PIM2	0.779	<0.001
IGLV4-3	0.778	<0.001
IGHV1-69	0.776	<0.001
IGLV1-50	0.776	<0.001
IGKV1D-13	0.774	<0.001
IGHV3-73	0.771	<0.001
IGHV3OR16-7	0.769	<0.001
ENAM	0.768	<0.001
IGKV1D-16	0.767	<0.001
IGHV3-52	0.765	<0.001
IGHV1-46	0.765	<0.001
IGLV2-8	0.765	<0.001
XBP1	0.765	<0.001
IGHV3-7	0.764	<0.001
AC016747.2	0.764	<0.001
IGKV1-16	0.762	<0.001
IGLV6-57	0.762	<0.001
IGHV4OR15-8	0.759	<0.001
WNT10B	0.756	<0.001
SFRP5	0.755	<0.001
IGHV1OR15-2	0.754	<0.001

TENT5C	0.750	<0.001
IGLV1-36	0.748	<0.001
IGHV1-69D	0.747	<0.001
AC104024.1	0.745	<0.001
IGHV3-72	0.744	<0.001
IGLV1-40	0.744	<0.001
IGLV3-16	0.743	<0.001
IGLV8-61	0.743	<0.001
AC244205.1	0.742	<0.001
IGHV3OR16-6	0.742	<0.001
CAMP	0.741	<0.001
IGHV2-26	0.740	<0.001
NPIPB15	0.738	<0.001
IGLV9-49	0.737	<0.001
ZBP1	0.735	<0.001
IGLC6	0.735	<0.001
TXNDC5	0.732	<0.001
CPNE5	0.732	<0.001
NLRP7	0.732	<0.001
PNOC	0.729	<0.001
IGHV3OR15-7	0.728	<0.001
LAX1	0.726	<0.001
IGKV2-30	0.726	<0.001
IGHV2-70	0.726	<0.001
IGKV1OR10-1	0.725	<0.001
AC016405.1	0.724	<0.001
IGLV11-55	0.724	<0.001
TMEM156	0.724	<0.001
AC012236.1	0.724	<0.001
KLK8	0.722	<0.001
AC007283.1	0.721	<0.001
BCO2	0.721	<0.001
C20orf197	0.721	<0.001
IGHV6-1	0.720	<0.001
TAS1R3	0.718	<0.001
IGHV3OR16-13	0.718	<0.001
IGHV2-70D	0.717	<0.001
IGLV5-48	0.715	<0.001
IGKV1D-27	0.715	<0.001
IGKV2D-26	0.712	<0.001
IGHV2-5	0.712	<0.001
AC022613.1	0.712	<0.001
STARD5	0.712	<0.001

AC012354.8	0.712	<0.001
IGLV4-60	0.711	<0.001
IGLVI-70	0.709	<0.001
AC136428.1	0.707	<0.001
IGHV3-38	0.707	<0.001
IGKV1OR2-118	0.702	<0.001
LINC01423	-0.701	<0.001
AL354710.2	-0.702	<0.001
AL353699.1	-0.702	<0.001
MAP2K3	-0.703	<0.001
GALNT14	-0.706	<0.001
NOC2LP1	-0.706	<0.001
HERC2P5	-0.706	<0.001
KRT17P5	-0.707	<0.001
AP006333.3	-0.707	<0.001
MPDU1	-0.709	<0.001
CYCS	-0.712	<0.001
HNRNPA1P14	-0.715	<0.001
AP000812.2	-0.715	<0.001
AL136038.4	-0.715	<0.001
AC073167.1	-0.715	<0.001
EEF1E1-BLOC1S5	-0.718	<0.001
SPA17	-0.718	<0.001
AGAP7P	-0.718	<0.001
AC019254.1	-0.719	<0.001
CHCHD2P6	-0.721	<0.001
MRRF	-0.721	<0.001
ATP5MC2	-0.721	<0.001
CABP1	-0.721	<0.001
AC126335.2	-0.722	<0.001
AC004917.1	-0.722	<0.001
AC079465.1	-0.722	<0.001
AC003965.1	-0.723	<0.001
AC093724.1	-0.724	<0.001
AL359697.1	-0.724	<0.001
RPS3AP33	-0.724	<0.001
AC004801.1	-0.726	<0.001
UBE3AP2	-0.726	<0.001
OR7E129P	-0.727	0.001
RPL26P35	-0.729	0.001
IGLL3P	-0.729	0.001
PCCA-DT	-0.729	0.002
MYH4	-0.732	0.001

SRPK3	-0.732	0.002
ENDOG	-0.732	0.002
AP001469.1	-0.732	0.002
HCFC1-AS1	-0.733	0.001
AC091965.4	-0.735	0.001
FBXL21P	-0.735	0.002
MKI67P1	-0.735	0.001
TP53TG3	-0.735	0.001
ZNF114	-0.738	0.002
KCNV1	-0.739	0.001
ZNF840P	-0.740	0.001
UQCRHL	-0.741	0.001
ATP5MG	-0.741	0.001
CCDC178	-0.741	0.001
AC139100.2	-0.741	0.001
RPL10P15	-0.741	0.001
CAPN13	-0.744	0.001
MRPS12	-0.744	0.001
AC140847.2	-0.745	0.001
MYH2	-0.746	0.001
PCDHA10	-0.747	0.001
AC131971.1	-0.747	0.001
DHRS7B	-0.747	0.001
TBC1D3P2	-0.750	0.001
LINC01119	-0.750	0.001
BEST3	-0.750	0.001
PSMD8	-0.750	0.001
AC005323.1	-0.751	0.001
AC068491.3	-0.752	0.001
APOBEC2	-0.753	0.001
AC004969.1	-0.753	0.001
TRPC3	-0.753	0.001
AL008628.1	-0.755	<0.001
AL359852.1	-0.755	<0.001
TP53TG3B	-0.756	<0.001
AC092447.5	-0.757	<0.001
AL513477.1	-0.759	<0.001
DMAC1	-0.759	<0.001
GNB5	-0.759	<0.001
AC138470.1	-0.759	<0.001
AC092850.1	-0.761	<0.001
SESTD1	-0.765	<0.001
C7orf25	-0.765	<0.001

TUBG1P	-0.765	<0.001
CR381670.1	-0.770	<0.001
TP53TG3F	-0.772	<0.001
AC073109.1	-0.774	<0.001
AP000439.2	-0.779	<0.001
AP003465.2	-0.782	<0.001
THAP12P8	-0.783	<0.001
AHCYP8	-0.785	<0.001
VPS25	-0.785	<0.001
MAPK8IP1P1	-0.788	<0.001
COPZ1	-0.791	<0.001
TAL2	-0.794	<0.001
MAPK8IP1P2	-0.797	<0.001
NRBF2P5	-0.800	<0.001
NSRP1P1	-0.803	<0.001
LRRC10B	-0.803	<0.001
RPS20P22	-0.808	<0.001
RN7SL656P	-0.815	<0.001
RN7SL199P	-0.815	<0.001
POLR1C	-0.818	<0.001
AC008764.4	-0.818	<0.001
AC108734.3	-0.826	<0.001
CBR1	-0.832	<0.001
TP53TG3E	-0.835	<0.001
C12orf50	-0.849	<0.001
AP000688.1	-0.862	<0.001
SLC25A15P3	-0.871	<0.001
PTPMT1	-0.874	<0.001

TCMR: T cell-mediated rejection; STA: stable renal function.

Supplementary Table 6. CPK-related genes in TCMR samples in GSE131179.

Gene	Correlation	*P* value
KDM2B-DT	-0.832	<0.001
SNORA18	-0.814	<0.001
KCNA3	-0.791	<0.001
ADAMTS7P4	-0.785	<0.001
UBE2FP1	-0.768	<0.001
Z97192.3	-0.765	<0.001
APOD	-0.753	0.001
SPIN2A	-0.753	0.001
RINL	-0.750	0.001
TNFSF8	-0.747	0.001
LDHAL6B	-0.743	0.001
F3	-0.741	0.001
AC009336.2	-0.741	0.001
AC087292.2	-0.738	0.002
TMEM130	-0.735	0.002
LINC02572	-0.726	0.001
AL121988.1	-0.726	0.001
BX679664.1	-0.724	0.002
AL583805.1	-0.719	0.002
AL683807.1	-0.718	0.002
TAS1R3	-0.715	0.003
BRI3P1	-0.710	0.002
AC007785.3	-0.708	0.002
SERPINI2	-0.705	0.002
HIPK4	-0.705	0.002
AC012020.1	-0.703	0.003
FER1L4	-0.703	0.003
MEI1	-0.703	0.003
AC113608.1	0.701	0.002
CR589904.1	0.702	0.002
AC084125.2	0.703	0.003
LINC00113	0.703	0.002
MYCN	0.706	0.003
LINC02028	0.706	0.003
FOLR1	0.706	0.003
AC026954.2	0.707	0.002
SLC22A3	0.709	0.003
MRPL32	0.709	0.003
NDUFA6	0.709	0.003
MT-ND1	0.709	0.003
AC046158.1	0.710	0.002

LINC01702	0.711	0.002
MROCKI	0.712	0.003
GPT	0.712	0.003
ENDOG	0.712	0.003
CLPB	0.712	0.003
AC048382.5	0.712	0.003
MRPS28	0.715	0.003
PLPPR5	0.715	0.002
EPHX2	0.718	0.002
CEL	0.718	0.002
YBX2	0.718	0.002
UGT1A4	0.718	0.002
MOGAT1	0.720	0.002
AC093904.4	0.721	0.002
SLIRP	0.721	0.002
LDHD	0.721	0.002
AL845331.2	0.722	0.002
AC024592.1	0.722	0.002
ADAM20	0.723	0.002
CCDC162P	0.724	0.002
NDUFB1	0.724	0.002
AC128707.1	0.726	0.001
AC099684.2	0.726	0.001
ASB17	0.726	0.001
ATP5MC3	0.726	0.002
PCCB	0.726	0.002
SULT1A2	0.726	0.002
SLC25A5	0.729	0.002
LINC01506	0.729	0.002
HMGN2P47	0.730	0.001
LINC02651	0.730	0.001
AP005431.2	0.732	0.001
AL450023.1	0.732	0.002
AC093151.2	0.734	0.001
AC243571.1	0.734	0.001
LDHBP2	0.735	0.002
PLIN5	0.735	0.002
B4GALT4-AS1	0.737	0.001
MAJIN	0.738	0.002
ACSM3	0.738	0.002
COX7CP1	0.744	0.001
BMS1P8	0.744	0.001
CHCHD4	0.747	0.001

ALDH1L1-AS2	0.747	0.001
RAC3	0.747	0.001
GCAT	0.747	0.001
AC006329.1	0.750	<0.001
CELP	0.750	<0.001
AC027612.1	0.753	<0.001
AL121895.1	0.753	<0.001
SLC25A15P1	0.754	<0.001
LINC02766	0.757	<0.001
AL109933.1	0.757	<0.001
HCFC1-AS1	0.758	<0.001
AC018659.6	0.761	<0.001
ATP5MC1	0.765	<0.001
AC018521.1	0.769	<0.001
ADIG	0.769	<0.001
GATB	0.771	<0.001
RB1-DT	0.776	<0.001
AC068533.1	0.783	<0.001
LINC00545	0.784	<0.001
MORN2	0.788	<0.001
PCCA-DT	0.788	<0.001
RCCD1	0.788	<0.001
BEST3	0.803	<0.001
OR7E129P	0.808	<0.001
AC135586.2	0.821	<0.001
AC003984.1	0.824	<0.001
ATP6V0E2-AS1	0.847	<0.001
AIMP2	0.871	<0.001

CPK: the number of clonotypes per thousand CDR3 reads; TCMR: T cell-mediated rejection; STA: stable renal function.

Supplementary Table 7. Differentially expression genes between TCMR and STA groups in GSE131179.

Gene	Log_2FC	Adjusted *P*
IGHG3	4.958	0.011
IGKV3-20	4.242	0.027
IGLC2	4.085	0.027
IGHV4-59	3.817	0.021
LYZ	3.649	0.001
AC020656.1	3.539	0.003
SLAMF7	3.337	0.021
CD3D	3.285	<0.001
TRBC2	3.261	0.003
GBP5	3.247	0.008
LCP1	3.130	<0.001
IL2RG	3.004	0.011
PTPRC	2.975	0.002
CD52	2.936	0.015
CD2	2.927	0.003
KLRK1	2.917	0.002
FCGR3A	2.911	<0.001
CTSS	2.896	<0.001
HLA-DQA1	2.860	0.004
CD8A	2.835	<0.001
LCK	2.736	0.003
CD3E	2.734	0.004
CD48	2.698	0.015
IGSF6	2.662	<0.001
VCAN	2.649	0.036
CD53	2.644	0.001
PLEK	2.615	0.006
TMC8	2.611	0.006
ITGAL	2.607	<0.001
CORO1A	2.604	0.003
C1QB	2.580	<0.001
TRAC	2.567	0.006
AC133555.4	2.561	<0.001
ITGAX	2.559	0.027
CALHM6	2.536	0.011
AC012645.3	2.528	0.015
MMP7	2.516	0.015
PARVG	2.487	<0.001
ARHGAP9	2.486	<0.001
LCP2	2.485	<0.001

JAML	2.477	0.036
LAPTM5	2.477	<0.001
PCED1B-AS1	2.460	0.004
HLA-DQB1	2.451	0.003
ITGB2	2.445	<0.001
NCKAP1L	2.427	<0.001
RAC2	2.426	<0.001
PTPRCAP	2.422	<0.001
CYBB	2.411	0.008
HCST	2.409	0.001
HLA-DQB1-AS1	2.408	0.036
SASH3	2.404	0.003
EVI2B	2.397	<0.001
FMNL1	2.371	0.001
MAP4K1	2.366	0.002
TYMP	2.360	<0.001
IL10RA	2.348	0.004
HLA-DPA1	2.328	0.001
JAK3	2.323	<0.001
SLC1A3	2.321	<0.001
MS4A6A	2.313	<0.001
IL2RB	2.312	0.002
RARRES1	2.307	0.015
HLA-DRA	2.303	<0.001
MYO1F	2.294	0.001
AOAH	2.278	0.001
CCR2	2.265	0.048
FGD2	2.261	<0.001
CD38	2.258	0.021
SEPTIN1	2.249	0.004
LTB	2.245	0.008
IL7R	2.245	0.015
IRF8	2.241	0.008
ARHGAP30	2.237	<0.001
CD247	2.235	0.011
LAIR1	2.233	<0.001
ZAP70	2.233	0.036
CCL4	2.223	0.048
NNMT	2.217	0.003
C1QC	2.214	<0.001
RASAL3	2.213	<0.001
ADCY7	2.212	<0.001
CD163	2.206	<0.001

PSTPIP1	2.205	0.002
SLAMF8	2.204	<0.001
LST1	2.204	0.048
C1orf162	2.202	<0.001
HLA-DOA	2.197	0.015
TBC1D10C	2.197	0.015
ACAP1	2.193	<0.001
MPEG1	2.177	0.003
C1QA	2.175	<0.001
FCGR1A	2.174	<0.001
HLA-DPB1	2.173	0.002
LILRB4	2.170	0.006
CD6	2.164	0.001
DOCK2	2.163	<0.001
PLCB2	2.152	<0.001
HLA-DMB	2.148	<0.001
THEMIS2	2.130	<0.001
IRF1	2.123	0.004
FCER1G	2.121	<0.001
HCLS1	2.120	<0.001
DGKA	2.109	<0.001
GBP2	2.108	<0.001
SH3BP1	2.105	<0.001
AL671883.2	2.103	<0.001
CXCR6	2.099	<0.001
TRIM22	2.099	0.011
FYB1	2.092	<0.001
ALOX5	2.087	<0.001
FCGR2C	2.084	0.027
LIMD2	2.084	0.015
PTPN7	2.079	0.015
BIRC3	2.077	<0.001
AC008105.3	2.066	0.036
SLA	2.065	0.001
CSF2RA	2.065	0.004
ITGB2-AS1	2.058	0.027
DEF6	2.055	0.002
VCAN-AS1	2.049	0.021
CD3G	2.046	0.008
BATF	2.041	<0.001
CD7	2.040	0.006
IL16	2.035	0.011
RHOH	2.026	0.008

SPI1	2.023	<0.001
ITGA4	2.023	0.021
S100A9	2.022	<0.001
SAMHD1	2.022	0.002
TYROBP	2.019	<0.001
CD37	2.016	<0.001
UCP2	2.016	0.036
ODF3B	2.015	0.003
CSF1R	2.012	0.015
DAPP1	2.003	<0.001
SNX20	1.999	0.021
SLAMF6	1.996	0.001
PIK3R5	1.987	0.001
RNASE6	1.981	<0.001
TAP1	1.978	0.004
BTK	1.974	<0.001
VAV1	1.972	<0.001
AL645939.2	1.965	0.008
NLRC5	1.965	0.004
EVI2A	1.962	0.001
MS4A7	1.962	<0.001
DOCK10	1.953	0.004
FPR3	1.951	<0.001
GPSM3	1.948	<0.001
CD27	1.947	0.002
CCDC88B	1.946	0.001
GZMM	1.944	0.011
BIN2	1.942	0.004
CCR5	1.941	0.001
IKZF1	1.941	<0.001
CD96	1.940	0.011
TGM2	1.940	0.048
LILRB1	1.937	0.015
CD86	1.936	<0.001
IRF4	1.933	0.002
CYTIP	1.932	0.006
SIT1	1.928	0.004
SECTM1	1.922	0.006
CD300A	1.918	<0.001
PSMB9	1.913	0.008
GZMH	1.911	<0.001
ADA2	1.908	0.048
ZBP1	1.896	0.027

SELPLG	1.895	0.004
CLEC2D	1.895	0.001
LGALS9	1.892	0.021
LY96	1.891	<0.001
HLA-DOB	1.888	0.036
SRGN	1.888	0.011
AKNA	1.885	<0.001
RASSF5	1.875	0.015
IL4I1	1.872	<0.001
MS4A4A	1.869	<0.001
HSH2D	1.862	<0.001
GBP4	1.858	0.027
MYO1G	1.848	0.003
TRAF3IP3	1.845	0.048
C3AR1	1.841	<0.001
VSIG4	1.835	<0.001
ITGAM	1.831	<0.001
CSF3R	1.831	<0.001
DOK2	1.830	0.001
FCGR1B	1.821	<0.001
APOBEC3G	1.820	0.003
RGS1	1.819	0.004
HCP5	1.817	<0.001
CLEC4E	1.817	0.008
PIM2	1.814	0.015
TNFSF13B	1.812	0.015
SAMSN1	1.809	0.001
NFKBIZ	1.805	0.015
TREM2	1.805	<0.001
CIITA	1.804	0.011
CXCR4	1.803	0.006
EMB	1.802	<0.001
ISG20	1.802	0.011
CD4	1.802	0.003
PPP1R18	1.795	<0.001
CARMIL2	1.792	0.001
INPP5D	1.788	0.011
GRAP2	1.785	0.027
CASP1	1.782	<0.001
GMIP	1.780	0.004
PTPN22	1.769	<0.001
KLRB1	1.767	0.048
HCK	1.765	0.003

HLA-DRB1	1.761	0.008
CARD11	1.760	<0.001
BTN3A1	1.755	0.001
CD84	1.743	<0.001
NCF1	1.740	0.002
MEI1	1.739	0.021
HLA-B	1.734	<0.001
PLA2G7	1.732	<0.001
SIRPG	1.731	0.027
APOL1	1.730	0.011
MRC1	1.730	0.006
LAT2	1.729	0.006
TMC6	1.727	0.006
MILR1	1.725	0.001
TNFAIP3	1.725	0.021
ARL4C	1.717	<0.001
FERMT3	1.717	0.003
NCF4	1.707	<0.001
BTN3A2	1.703	0.021
CYTH4	1.701	0.011
CXCL1	1.700	0.006
CD5	1.698	0.001
DHRS9	1.695	0.006
CELF2	1.694	0.015
HLA-F	1.691	0.015
CXCR3	1.691	0.008
RUNX3	1.691	0.008
FCRL6	1.690	0.003
TAGAP	1.685	0.008
PARP14	1.685	0.015
WAS	1.685	0.011
IFI16	1.677	0.011
FCGR2A	1.676	<0.001
APBB1IP	1.674	0.011
RHBDF2	1.673	0.002
PSMB8-AS1	1.672	0.001
AC243960.1	1.671	<0.001
FGR	1.671	0.006
SPN	1.666	0.004
ITK	1.665	0.002
MSR1	1.665	0.006
AL671277.1	1.647	<0.001
TNFRSF1B	1.636	0.036

CSF2RB	1.634	0.004
SOCS3	1.633	0.011
ZNF683	1.630	0.036
CD33	1.626	0.008
CARD16	1.626	0.006
GLIPR1	1.625	0.027
TNFRSF12A	1.624	0.027
FXYD5	1.621	0.004
AL121985.1	1.617	0.011
APOBEC3D	1.617	0.001
SLFN12L	1.613	0.006
ARPC1B	1.611	0.004
GIMAP2	1.609	<0.001
TNFAIP8	1.608	0.004
PRF1	1.607	0.048
HLA-DMA	1.604	<0.001
PYHIN1	1.602	0.002
SIGLEC1	1.602	0.001
NCF1B	1.601	0.001
TCIRG1	1.600	0.021
PATL2	1.595	0.008
P2RX5	1.594	0.036
LMNB1	1.592	0.021
JUNB	1.591	<0.001
IL12RB1	1.587	<0.001
MARCHF1	1.581	0.011
AIM2	1.581	0.015
TRPM2	1.579	0.004
B2M	1.578	<0.001
EMILIN2	1.575	0.036
LY86	1.575	0.001
PYCARD	1.573	<0.001
CLEC12A	1.570	0.008
SCIMP	1.569	0.015
UNC13D	1.565	0.021
FCGR3B	1.561	<0.001
SP140	1.560	0.002
APOL3	1.560	0.027
CFB	1.559	0.001
BTN3A3	1.559	0.003
LILRB2	1.554	0.036
RGS10	1.553	0.036
GIMAP4	1.548	0.001

FAM78A	1.546	<0.001
ZNF385A	1.546	0.027
MDK	1.539	0.008
SYTL1	1.539	<0.001
ICAM1	1.539	0.004
SLFN11	1.538	0.015
SLC37A2	1.535	0.004
STK17B	1.532	<0.001
PARP15	1.532	<0.001
NAIPP3	1.531	<0.001
NAIPP1	1.527	0.001
IKZF3	1.526	0.004
EBI3	1.525	0.027
TAP2	1.525	0.004
RASSF2	1.512	0.015
LY9	1.508	0.036
CCL3	1.507	0.027
RNF213	1.507	0.001
CD74	1.506	<0.001
SIGLEC10	1.503	0.048
PLEKHO1	1.503	0.036
AC008105.1	1.500	<0.001
STK10	1.495	0.004
NFE2L3	1.486	0.036
SDC3	1.484	0.011
NCF1C	1.483	0.002
BCL3	1.481	<0.001
HLA-A	1.479	0.001
PSTPIP2	1.478	0.003
UBASH3A	1.477	0.008
BAZ1A	1.474	<0.001
RUNX1	1.473	0.003
PARP8	1.469	0.003
FCHO1	1.469	0.003
DTX3L	1.461	<0.001
GMFG	1.455	0.048
NFKBID	1.450	0.008
CD79B	1.450	0.002
GPNMB	1.449	0.048
DOK3	1.446	0.004
SH2D1A	1.445	0.002
CD69	1.441	0.003
UBE2L6	1.439	0.006

SAMD9L	1.438	0.006
AC004151.1	1.436	<0.001
CLDN1	1.434	0.015
FCRL3	1.429	0.048
SMAP2	1.428	<0.001
PTAFR	1.422	<0.001
LAG3	1.422	0.011
TNFAIP8L2	1.421	0.006
FPR1	1.419	0.011
PLAUR	1.418	0.008
STK17A	1.417	0.004
IL27RA	1.416	0.004
GGTA1P	1.413	0.011
APOBEC3C	1.410	0.008
ARHGAP25	1.410	0.004
KLHL6	1.407	0.003
LPAR2	1.402	0.011
MCUB	1.402	<0.001
CD1D	1.401	0.004
SOD2	1.399	0.027
ARHGDIB	1.398	0.011
WIPF1	1.397	0.003
STAT4	1.395	<0.001
CP	1.392	0.027
HLA-J	1.392	0.036
ANXA1	1.389	0.008
IL10RB-DT	1.386	0.002
TLR2	1.383	<0.001
SLAMF1	1.382	0.011
CMTM3	1.378	0.004
SLC15A3	1.378	0.036
HLA-C	1.377	0.003
APOL6	1.376	<0.001
ALOX5AP	1.376	0.036
RGS19	1.376	0.036
LRRK1	1.373	0.003
FCGR1CP	1.368	<0.001
TMSB4XP8	1.366	0.036
LPXN	1.366	<0.001
CXCL16	1.364	0.036
PARP9	1.364	0.003
CLEC2B	1.361	0.003
FOLR2	1.357	0.027

AL365361.1	1.356	<0.001
TNFAIP2	1.353	<0.001
TESPA1	1.352	0.036
PSMB8	1.351	0.001
IFNAR2	1.349	<0.001
SH2D2A	1.347	0.004
CLIC2	1.345	0.002
BTN2A2	1.344	0.011
P2RY6	1.342	0.015
GNG2	1.340	0.027
ARID5A	1.340	0.011
CASP8	1.339	<0.001
EOMES	1.338	0.003
ENAM	1.333	0.036
LINC00861	1.322	0.015
GPR183	1.322	0.001
AGAP2	1.322	<0.001
LAMP3	1.321	0.015
BCAS4	1.321	0.048
FAM49B	1.319	0.004
TNFSF8	1.315	0.006
RESF1	1.305	0.001
TLR7	1.305	0.004
P2RY13	1.298	0.008
TAPBP	1.297	0.048
CCL4L2	1.297	0.015
EVL	1.296	0.011
AL031595.1	1.295	<0.001
TMIGD3	1.294	<0.001
ARHGAP4	1.290	0.011
NFKB2	1.284	0.004
PSMB10	1.284	0.021
RELT	1.283	<0.001
MYO9B	1.283	0.004
RIPOR2	1.279	0.008
RNF166	1.276	0.048
ANXA2P2	1.273	0.011
ATF3	1.271	<0.001
FER1L4	1.268	0.015
ADA	1.267	<0.001
MLKL	1.266	<0.001
KCTD17	1.266	0.021
SIRPB2	1.263	0.015

LRRC25	1.262	0.011
CARD9	1.259	0.004
CEACAM21	1.258	<0.001
CD300LF	1.257	0.021
RELB	1.255	0.003
CYTOR	1.253	0.001
MFNG	1.252	0.011
S100A11	1.246	0.021
BTG2	1.244	0.004
ARRDC2	1.243	0.011
FAM49A	1.243	0.036
RIPK3	1.242	0.036
TRIM69	1.238	0.021
PLEKHO2	1.236	<0.001
PIK3CG	1.234	0.011
ABCA7	1.234	0.048
LAX1	1.229	0.008
RRM2	1.229	0.036
SCML4	1.226	<0.001
GRK2	1.226	0.048
TNRC6C-AS1	1.225	0.011
LHFPL2	1.220	0.004
HLA-E	1.219	<0.001
SP110	1.219	0.008
CASP4	1.216	<0.001
TRAF1	1.212	0.003
PIK3CD	1.211	0.008
TTC39C	1.211	<0.001
MIAT	1.210	0.021
MYC	1.208	0.015
NLRP1	1.204	0.015
GPR34	1.204	<0.001
IRF9	1.202	0.036
GAPT	1.202	0.036
RUBCNL	1.202	0.027
MFSD2A	1.202	0.006
SLC16A3	1.199	0.027
ITGB3	1.198	0.048
NLRC3	1.197	0.003
APOL2	1.193	0.008
OASL	1.191	0.003
C16orf54	1.189	0.011
HSPA6	1.189	<0.001

CDC42SE2	1.187	0.006
TNF	1.178	0.003
CXCL6	1.176	0.002
GAB3	1.174	0.001
PREX1	1.173	0.001
RNASET2	1.173	0.027
CDKN1A	1.173	0.036
NFKBIE	1.172	<0.001
TBXAS1	1.171	0.036
AC007620.2	1.170	0.027
MAP3K8	1.169	0.021
ARMH1	1.166	0.021
ZC3H12A	1.161	0.006
ACTR3	1.160	0.008
AC133065.3	1.159	<0.001
GIMAP7	1.159	0.008
ANKRD13D	1.158	0.008
TLR1	1.157	0.006
NFATC2	1.157	0.011
KLF10	1.155	0.027
APOBEC3F	1.149	<0.001
SLFN5	1.148	0.036
MARCO	1.145	0.048
SLC9A9	1.142	<0.001
PLK3	1.142	0.002
HMGA1	1.139	0.021
CEBPB	1.138	<0.001
STK4	1.137	0.011
LIF	1.136	0.008
SNHG12	1.131	0.015
CD40	1.130	0.027
IKBKE	1.129	0.003
P2RY8	1.129	0.006
AP000763.4	1.125	0.003
APOBR	1.125	0.015
PDCD1LG2	1.123	0.003
GPR65	1.120	<0.001
PTPN6	1.120	<0.001
ARNTL2	1.116	0.027
GFI1	1.113	0.002
SOX9	1.112	<0.001
IFNGR1	1.112	0.008
TRAT1	1.110	0.036

ADAM19	1.106	0.002
ST8SIA4	1.104	0.008
TRANK1	1.102	0.002
KLRG1	1.101	0.036
PRDM1	1.100	<0.001
MCL1	1.097	0.003
HVCN1	1.095	0.036
DDX60L	1.094	0.036
RTP4	1.093	0.003
CRTAM	1.092	<0.001
MIDN	1.092	0.027
AC104758.1	1.088	0.006
MSL3	1.085	0.048
TLDC2	1.083	0.004
CYLD	1.082	0.003
MYD88	1.081	0.002
LRG1	1.081	<0.001
TLR8	1.081	0.002
AC011511.5	1.080	0.004
VNN2	1.076	0.036
THEMIS	1.075	<0.001
ELF4	1.074	0.027
NFAM1	1.073	<0.001
APOBEC3H	1.064	0.048
NCR3	1.064	0.008
ZFP36	1.063	0.008
UNC93B1	1.062	0.008
CASP3	1.061	0.006
CD72	1.061	0.011
GPR18	1.060	0.004
PRAM1	1.060	0.048
BATF3	1.060	0.015
NRROS	1.058	0.036
TNK2	1.058	<0.001
PPM1M	1.056	0.027
RUFY4	1.055	0.027
PCED1B	1.054	0.048
KIF21B	1.052	0.002
LIMK1	1.050	0.015
IL18BP	1.048	0.011
IL10RB	1.048	0.048
PPP1R9B	1.048	0.015
ZDHHC18	1.045	0.008

FAS	1.044	0.021
EFHD2	1.041	0.004
NMI	1.041	0.001
DENND2D	1.030	0.021
CSK	1.030	0.048
MMP25-AS1	1.028	0.003
TPM3	1.022	0.004
SYTL3	1.015	<0.001
DUX4L50	1.014	0.004
CD47	1.012	0.027
CXorf21	1.011	0.001
ARHGEF1	1.010	0.036
DYRK2	1.010	0.021
ZNF267	1.009	0.036
SIGLEC9	1.009	0.002
LILRA6	1.007	0.002
TMEM154	1.005	0.003
MST1R	1.005	0.036
MAFB	1.003	0.011
C9orf139	0.999	0.006
PIK3R6	0.997	<0.001
PLEKHF1	0.996	0.001
ADAM28	0.993	0.015
CBLN3	0.993	0.006
RHOG	0.991	0.021
EZH2	0.990	0.027
KIAA0930	0.989	0.002
TNFSF14	0.987	<0.001
HPSE	0.985	<0.001
GNA15	0.985	0.004
GRK6	0.984	0.036
PIF1	0.973	0.006
SH3BP2	0.971	0.036
MAP3K14-AS1	0.970	0.027
SERPINB1	0.968	0.003
HECA	0.968	0.015
SFMBT2	0.968	0.021
STAMBPL1	0.967	0.002
ETV6	0.966	0.011
GCSAM	0.966	0.002
CHFR	0.965	0.036
TEP1	0.964	0.003
SLA2	0.962	0.027

CXCL2	0.960	0.036
ARRB2	0.960	0.001
CD19	0.954	0.027
MYO5A	0.951	0.027
CARD8-AS1	0.950	0.036
STAT1	0.949	0.021
FASLG	0.949	<0.001
TRIM21	0.944	0.015
SIGLEC7	0.944	0.036
TBX21	0.942	0.021
NCK1	0.941	0.011
REEP4	0.934	0.011
RAP2B	0.931	0.002
MIR3142HG	0.931	0.036
CD180	0.931	<0.001
SAMD9	0.930	0.021
DUSP6	0.930	0.036
CYBC1	0.929	0.008
HK2	0.926	0.011
ARPC5	0.924	0.004
OSCAR	0.921	0.006
ITGB7	0.919	0.008
AC011446.2	0.917	0.027
AD000864.1	0.914	<0.001
MAFF	0.911	0.004
GRK3	0.911	0.015
WAKMAR2	0.907	<0.001
PML	0.906	0.015
UGCG	0.903	<0.001
SIGLEC14	0.900	0.048
ZMYND15	0.899	0.008
NIN	0.890	0.015
CGAS	0.889	0.002
DPYD	0.877	0.027
ZNF101	0.874	<0.001
SLC1A4	0.869	0.002
MS4A4E	0.864	0.015
DGKQ	0.859	0.015
AC025423.4	0.858	0.027
RABGAP1L	0.857	0.036
BLM	0.845	0.027
CCR4	0.844	0.008
ARL6IP5	0.843	0.021

CORO7	0.843	0.004
ZC3HAV1	0.842	0.021
ZFP36L2	0.841	0.048
LYAR	0.841	0.021
LINC01943	0.833	0.011
FANCA	0.833	0.027
Z97986.1	0.833	0.015
RHOU	0.829	0.004
AL133453.1	0.827	0.036
ANXA2P1	0.827	<0.001
IL1RN	0.826	0.015
RNASE2	0.824	0.028
AC046185.3	0.821	0.008
IL21R-AS1	0.817	0.006
DUSP5	0.815	0.006
LINC01138	0.814	0.003
NECAP2	0.813	0.006
RIPK2	0.813	0.008
SH3KBP1	0.812	0.015
FBXO41	0.811	0.002
AC007068.1	0.810	0.001
CFLAR	0.810	0.048
AC026462.1	0.808	0.015
P2RY12	0.807	<0.001
SCN1B	0.805	0.021
CCDC88C	0.804	0.048
RASSF1	0.803	0.027
BCL11B	0.801	0.027
MYCL	0.800	0.048
RAP1AP	0.798	0.048
TRAF2	0.796	0.015
NBPF8	0.790	0.004
MAP3K14	0.788	<0.001
CD300C	0.787	<0.001
CYSLTR1	0.786	0.004
MOB3A	0.785	0.021
XBP1	0.785	0.002
PHLDA2	0.781	0.015
LINC00869	0.779	0.011
TRIM38	0.778	<0.001
TNIK	0.774	0.008
BMP2K	0.774	0.048
NUP62	0.774	0.036

HMGN1P38	0.772	0.021
ANXA2R	0.772	<0.001
LACC1	0.770	<0.001
IFNGR2	0.769	0.011
Z99774.1	0.768	0.015
MGAT1	0.766	0.004
STAT5A	0.766	0.048
H2AX	0.765	0.036
ASCC3	0.763	0.008
CCSAP	0.760	0.036
CMTM7	0.760	0.002
ARF6	0.758	0.027
CCL3L3	0.757	0.004
C6orf62	0.754	0.021
CD200R1	0.752	0.027
APOBEC3B	0.746	0.002
DUSP10	0.745	0.001
LINC00623	0.738	0.048
PTPN1	0.735	0.036
COL8A2	0.730	0.006
TRABD	0.727	0.027
AC012615.3	0.725	0.006
SMARCA5	0.721	0.036
OGFR	0.716	0.036
ARPC2	0.714	0.021
RASSF1-AS1	0.712	0.027
AP4B1-AS1	0.710	0.008
PIWIL4	0.703	0.008
AEN	0.703	0.003
SNAI3	0.696	0.011
AC005332.4	0.695	<0.001
CDC45	0.694	0.003
SHTN1	0.693	0.027
IER2	0.692	0.006
SOWAHD	0.689	0.027
TMEM268	0.687	0.027
BORA	0.687	0.015
AKAP13	0.674	0.036
TLR6	0.673	<0.001
MARS1	0.673	0.036
CRACR2A	0.666	0.006
CCNA2	0.653	0.036
C1orf131	0.650	0.027

MELK	0.647	0.036
SLC35C1	0.646	0.036
EIF4A1	0.643	0.036
TOR3A	0.641	0.027
TUBAP2	0.638	0.048
RPL22L1	0.636	0.011
NPM1	0.635	0.015
RAC1P2	0.635	0.021
NAGK	0.635	0.011
MITD1	0.635	0.048
AC026877.1	0.633	0.008
CCR6	0.632	0.015
NCMAP	0.625	<0.001
SRSF7	0.624	0.048
LYSMD2	0.622	0.027
M6PR	0.621	0.027
EIF4A1P10	0.614	0.048
UHRF1	0.609	0.008
GVINP1	0.608	0.011
KCNA3	0.601	0.049
ORAI1	0.598	0.021
AC010186.3	0.582	0.011
SIGLEC16	0.577	0.027
TRIM27	0.575	0.036
AC011899.2	0.563	0.015
EIF4A1P2	0.558	0.004
AC021739.2	0.555	0.008
AC104966.1	0.554	0.015
ERMN	0.550	0.021
NLRC4	0.545	0.048
ZUP1	0.544	0.027
AC006441.3	0.536	0.015
TRNAU1AP	0.532	0.048
LINC00528	0.529	0.001
FUT4	0.524	<0.001
AL139099.2	0.523	0.036
RAN	0.522	0.036
ADRB2	0.517	<0.001
ADGRE4P	0.514	0.004
RAB33A	0.512	0.004
AC006978.1	0.512	0.001
ARPC3	0.507	0.021
PABPC3	0.505	0.004

PPIH	0.500	0.015
ZPR1	0.497	0.036
TNFRSF11A	0.496	0.004
NRBF2	0.495	0.036
TNFRSF13C	0.488	0.015
AP002358.2	0.477	0.036
Z83844.2	0.451	0.048
SACS	0.448	0.015
HNRNPA1P39	0.447	0.021
AC241585.2	0.436	0.048
CLEC5A	0.434	0.027
LINC00672	0.407	0.036
YIPF1	0.401	0.036
ARL11	0.387	<0.001
ALDH1L2	0.371	0.011
UNC93B7	0.368	0.036
HELB	0.364	0.006
MEFV	0.357	0.003
DAP3	0.351	0.027
NGLY1	0.340	0.008
RAB39B	0.332	0.015
AC022075.1	0.325	0.048
HSPB11	0.324	0.011
TMEM121B	0.316	0.011
CCR8	0.306	0.018
LGALS9C	0.299	0.004
LRFN1	0.292	0.006
TXLNB	0.268	0.036
LINC002481	0.260	0.021
BLACAT1	0.233	0.027
AL663070.2	0.228	0.015
AC105749.1	0.224	0.036
C1orf127	0.219	0.036
RPSAP45	0.203	0.036
FAM225A	0.200	0.004
FAM225B	0.181	0.027
HS3ST2	0.169	0.027
ZNF469	0.169	0.015
NPFFR1	0.123	0.035
NLRP7	0.116	0.003
RIMBP3	0.086	<0.001
RIMBP3C	0.072	<0.001
RIMBP3B	0.068	0.004

PMVK	-0.434	0.003
GSTM4	-0.527	0.048
AC018521.5	-0.563	0.027
HINT2	-0.614	0.021
AGBL4	-0.632	0.021
CENPS	-0.722	0.011
MTND1P23	-0.751	0.027
MT-ATP6	-0.792	0.036

TCMR: T cell-mediated rejection; STA: stable renal function; LogFC: log fold change.

Supplementary Table 8. Expression of *MEI1* across all cell populations in the Kidney Tissue Atlas.

Cluster abbreviation	Cluster Name	Cell Count	Mean Expression	Cells Expressing	Fold Change	*P*	Adjusted *P* -values
PL	Plasma Cell	406	0.425	27.8	0.473	< 0.0001	< 0.0001
EC-AEA	Afferent / Efferent Arteriole Endothelial Cell	1748	0.0302	NS	NS	NS	NS
B	B Cell	1148	0	NS	NS	NS	NS
cDC	Classical Dendritic Cell	1708	0.036	NS	NS	NS	NS
CNT	Connecting Tubule Cell	3217	0	NS	NS	NS	NS
dCNT	Connecting Tubule Cell	1864	0.00231	NS	NS	NS	NS
CNT-IC-A	Connecting Tubule Intercalated Cell Type A	1988	0	NS	NS	NS	NS
CNT-PC	Connecting Tubule Principal Cell	549	0.00317	NS	NS	NS	NS
dCNT-PC	Connecting Tubule Principal Cell	3125	0.00403	NS	NS	NS	NS
C-TAL	Cortical Thick Ascending Limb Cell	15850	0.00317	NS	NS	NS	NS
dC-TAL	Cortical Thick Ascending Limb Cell	1923	0	NS	NS	NS	NS
T-CYT	Cytotoxic T Cell	947	0.337	NS	NS	NS	NS
DTL1	Descending Thin Limb Cell Type 1	1719	0.00153	NS	NS	NS	NS
dDCT	Distal Convoluted Tubule Cell	1600	0	NS	NS	NS	NS
DCT1	Distal Convoluted Tubule Cell Type 1	3289	0.000678	NS	NS	NS	NS
cycEC	Endothelial Cell	39	0.0288	NS	NS	NS	NS

cycEPI	Epithelial Cell	219	0	NS	NS	NS	NS
FIB	Fibroblast	720	0.0526	NS	NS	NS	NS
aFIB	Fibroblast	178	0	NS	NS	NS	NS
EC-GC	Glomerular Capillary Endothelial Cell	1426	0.006	NS	NS	NS	NS
IC-A	Intercalated Cell Type A	3352	0	NS	NS	NS	NS
dIC-A	Intercalated Cell Type A	429	0.00204	NS	NS	NS	NS
IC-B	Intercalated Cell Type B	673	0.00453	NS	NS	NS	NS
EC-LYM	Lymphatic Endothelial Cell	105	0.0396	NS	NS	NS	NS
MAC-M2	M2-Macrophage	892	0.0435	NS	NS	NS	NS
MAST	Mast Cell	31	0.0216	NS	NS	NS	NS
M-TAL	Medullary Thick Ascending Limb Cell	1074	0.00182	NS	NS	NS	NS
MC	Mesangial Cell	130	0.0149	NS	NS	NS	NS
MON	Monocyte	1329	0.375	NS	NS	NS	NS
MDC	Monocyte-derived Cell	1332	0.0342	NS	NS	NS	NS
cycMNP	Mononuclear Phagocyte	209	0.263	NS	NS	NS	NS
MyoF	Myofibroblast	76	0.0242	NS	NS	NS	NS
NK1	Natural Killer Cell Type 1	791	0.266	NS	NS	NS	NS
NK2	Natural Killer Cell Type 2	206	0.126	NS	NS	NS	NS
NKT	Natural Killer T Cell	1252	0.223	NS	NS	NS	NS
ncMON	Non-classical Monocyte	367	0.00682	NS	NS	NS	NS
PEC	Parietal Epithelial Cell	631	0.00712	NS	NS	NS	NS
EC-PTC	Peritubular Capillary Endothelial Cell	4229	0	NS	NS	NS	NS
dEC-PTC	Peritubular Capillary Endothelial Cell	262	0	NS	NS	NS	NS
pDC	Plasmacytoid Dendritic Cell	207	0.0708	NS	NS	NS	NS

POD	Podocyte	244	0	NS	NS	NS	NS
PC	Principal Cell	4801	0.000477	NS	NS	NS	NS
dPC	Principal Cell	2335	0.00214	NS	NS	NS	NS
tPC-IC	Principal-Intercalated Cell	1687	0.022	NS	NS	NS	NS
aPT	Proximal Tubule Epithelial Cell	9878	0.0012	NS	NS	NS	NS
dPT	Proximal Tubule Epithelial Cell	5787	0.00277	NS	NS	NS	NS
dPT/DTL	Proximal Tubule Epithelial Cell / Descending Thin Limb Cell	2423	0.00128	NS	NS	NS	NS
PT-S1/S2	Proximal Tubule Epithelial Cell Segment 1 / Segment 2	7041	0	NS	NS	NS	NS
PT-S3	Proximal Tubule Epithelial Cell Segment 3	181	0	NS	NS	NS	NS
T-REG	Regulatory T Cell	241	0.318	NS	NS	NS	NS
REN	Renin-positive Juxtaglomerular Granular Cell	168	0	NS	NS	NS	NS
T	T Cell	4359	0.185	NS	NS	NS	NS
cycT	T Cell	232	0.425	NS	NS	NS	NS
aTAL1	Thick Ascending Limb Cell Cluster 1	1023	0.00771	NS	NS	NS	NS
aTAL2	Thick Ascending Limb Cell Cluster 2	6629	0	NS	NS	NS	NS
dVSMC	Vascular Smooth Muscle Cell	849	0.0381	NS	NS	NS	NS
VSMC/P	Vascular Smooth Muscle Cell / Pericyte	1228	0	NS	NS	NS	NS
